# Stepwise Reactions in the Potassium and Ammonia-Intercalated Iron Selenide Superconductor Phase Diagram Followed by *In Situ* Powder Diffraction

**DOI:** 10.1021/jacs.5c00356

**Published:** 2025-05-19

**Authors:** Simon J. Cassidy, Daniel N. Woodruff, Stefan J. Sedlmaier, Jack N. Blandy, Christina Reinhard, Oxana V. Magdysyuk, Andrew L. Goodwin, Silvia Ramos, Simon J. Clarke

**Affiliations:** † Department of Chemistry, 6396 Inorganic Chemistry Laboratory, University of Oxford, South Parks Road, Oxford OX1 3QR, U.K.; ‡ 120796 Diamond Light Source Ltd, Harwell Science and Innovation Campus, Didcot OX11 0DE, U.K.; § The University of Manchester at Harwell, Diamond Light Source, Harwell Campus, Didcot, Oxfordshire OX11 0DE, U.K.; ∥ EaStCHEM, School of Chemistry, 7486 University of St Andrews, North Haugh, St Andrews KY16 9ST, U.K.; ⊥ School of Physics and Astronomy, 2240 University of Kent, Canterbury CT2 7NH, Kent, U.K.

## Abstract

Iron-based superconductors have attracted much attention for their high superconducting temperatures and high upper critical fields, which make them promising candidates for application as well as fundamentally important for our understanding of superconductivity. One feature of these superconductors is their ability to intercalate and deintercalate species from between their iron-containing layers, something not available in cuprate high-temperature superconductors or niobium-based conventional superconductors used in technologies. This provides an opportunity for switchable changes in the superconducting properties as a function of chemical conditions, but the resulting structures are often hard to characterize due to loss of crystallinity and sometimes the formation of multiphase products. Here, we explore both the synthesis and decomposition of potassium and ammonia-intercalated iron selenide superconductors through *in situ* powder X-ray diffraction. We report a complete phase diagram including two new solution-stable ammonia-rich phases and several metastable forms. We give accurate characterization of the reported ammonia-poor forms using a combination of neutron and X-ray powder diffraction, using an innovative supercell approach to describe the phase breadth within the samples. These results give rare insight into stepwise changes occurring in solids along multiple reaction pathways, which demonstrate the importance of *in situ* diffraction techniques.

## Graphical abstract

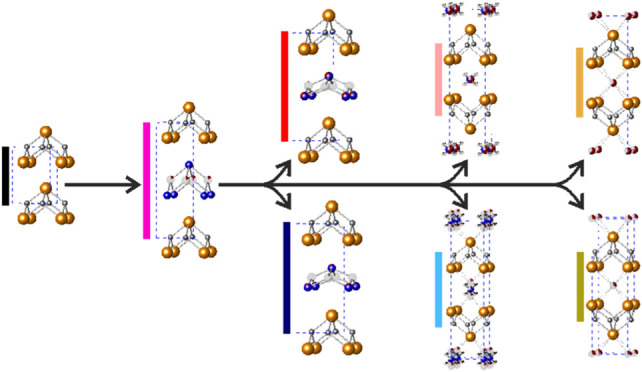

## Introduction

The iron-based superconductor (IBS) family sparked great excitement upon its discovery and continues to inspire a wide range of research.^1,2^ Bulk superconductivity in IBSs can reach transition temperatures (*T*_c_) as high as 55 K in SmO_1–*x*_F_*x*_FeAs and they can have upper critical fields (*H*_*c*2_) that exceed 50 T in LaO_1–*x*_H_*x*_FeAs;^3,4^ records far exceeding those of the niobium-based superconductors (Nb–Ti alloys and Nb_3_Sn) used in most applications today. Challenges in processing these ceramics for application are gradually being overcome with wires that can hold critical current densities above 71 kA cm^–2^ in Ba_1–*x*_Na_*x*_Fe_2_As_2_ and superstrength permanent magnets (2.83 T) of Ba_1–*x*_K_*x*_Fe_2_As_2_ entering the literature.^5,6^

Many branches of the IBS family have been discovered, each containing anti-PbO type layers of Fe­(*Ch*/*Pn*), where *Ch* is a chalcogenide or *Pn* a pnictide, which are central to the superconducting mechanism. Archetypal FeSe contains only these superconducting layers resulting in desirable topological properties, with tellurium-doped Fe­(Se,Te) reportedly showing topological insulator, topological Dirac semimetal, and topological superconductor states in its phase diagram,^7,8^ properties which are highly sought after in the field of quantum computing.^9^

The layered nature of the IBSs provides an opportunity for low-temperature chemical modification to alter the superconducting properties without altering the layer topology.^10^ This was demonstrated by Ying et al. with the discovery that lithium and other alkali, alkaline earth, and rare earth elements would intercalate into β-FeSe and provide a large increase in *T*_c_ when these elements were dissolved in liquid ammonia.^11,12^ The chemical space has proven even more expansive than originally thought by the existence of “ammonia-rich” phases, which are formed in solution but exhale some of the intercalated ammonia when removed from an ammonia-rich environment to produce relatively “ammonia-poor” phases which can be handled under inert conditions at ambient temperatures and pressures.^13,14^ Reports on the ammonia-rich phases utilize *in situ* characterization under ammonia atmospheres or in liquid ammonia to get a true picture of the phases formed and the reaction pathways between them.^13,14^

A forerunner of the low-temperature-intercalated iron selenides was the high-temperature-synthesized K_*x*_Fe_2–*y*_Se_2_, where *x* ≈ 0.8 and *y* ≈ 0.4, which was originally reported to exhibit superconductivity despite high concentrations of vacancies on the iron site.^15^ It was later shown to contain an iron-vacancy-free phase of approximate composition K_0.2_Fe_2_Se_2_ with average oxidation state Fe^1.9+^, which forms superconducting filaments within the antiferromagnetic insulating bulk phase of K_2_Fe_4_Se_5_ (Fe^2+^).^16,17^ A lesson to be learned here is that slight reduction of iron below +2 promotes the superconducting state in these systems but it must be finely controlled to prevent further reduction to Fe(0).^18–21^ In this role, the cointercalation of ammonia can provide a neutral molecule to aid the separation of the layers without requiring high concentrations of the reducing metal that lead to over-reduction. Close examination of deuterium occupancies in the neutron diffraction patterns of the lithium/ammonia intercalates of iron selenide reveals that ammonia can be redox active in the synthesis, with ammonia partially reduced to amide (and H_2_ evolved) which cointercalates with the metal and ammonia in the layers, further lowering the amount to which Fe is reduced.^12,13^ This reduction buffer provided by ammonia allows many metals of varying reduction potential to produce stable and superconducting phases by cointercalation into iron selenide layers.^10,11^

Characterization of the potassium and ammonia-intercalated iron selenide by Ying et al. shows these are similar to those of the ammonia-poor lithium intercalates,^22^ except the authors demonstrate a phase gap that opens between stoichiometries using 0.15 and 0.3 mol of K per mole of FeSe in the intercalation reactions, giving superconducting phases with different *T*_c_s. The phases on these two sides of the phase gap have very similar structures but differ significantly in the separation between the FeSe layers, approximately 7.9 and 7.4 Å, respectively. The authors go on to show that thermal treatment of the phase with a stoichiometry of 0.15 K/FeSe can result in an ammonia-free version of the intercalate with retention of the superconducting properties.

In this extensive report, we look at the full phase diagram of potassium/ammonia intercalates of iron selenide, as it is given in Figure 1. This figure displays each phase that we will discuss and refer back to throughout the report, and shows the pathway(s) to the formation of each one from the iron selenide starting material. Our results and discussion will be divided into three subsections to address the following three classes of potassium and ammonia-intercalated iron selenide compounds in turn:

- Ammonia-rich: we explore their synthesis via *in situ* X-ray Powder diffraction (XRPD) and provide the first characterization of two ammonia-rich forms of the intercalate products and a crystalline intermediate.
- Ammonia-poor: we use high-resolution, synchrotron X-ray and neutron-powder diffraction (NPD) to provide an accurate description of the structures of the ammonia-poor forms already identified in the literature and give an explanation for the phase gap observed.^22^
- Ammonia-free: we use *in situ* XRPD to probe the thermal treatment of the intercalates, which results in the loss of ammonia and the formation of phases analogous to the high-temperature-synthesized K_*x*_Fe_2–*y*_Se_2_ phases.

**Figure 1:**
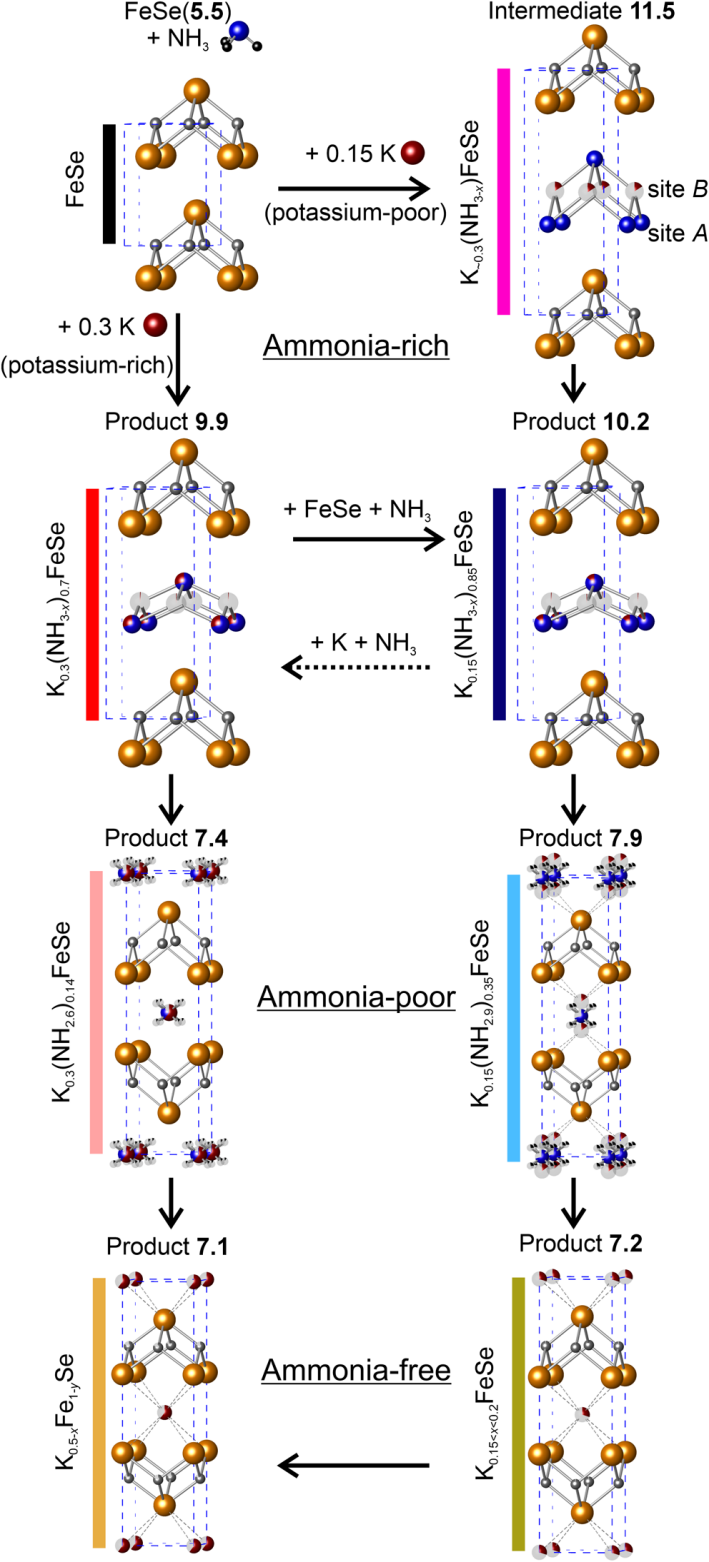
Potassium and ammonia-intercalated iron selenide phases. Each phase is given a product or intermediate number, which corresponds to its approximate interlayer separation. Colors that are used throughout the paper to identify each phase are shown alongside the assigned composition of each phase determined in this work. The dotted arrow between **10.2** and **9.9** indicates that this process is inferred but has not been observed.

In each subsection of the report, we will discuss both branches of the phase diagram, which are accessed by reaction with either a potassium-rich K/FeSe molar ratio (0.3:1, left side of Figure 1) or a more potassium-poor (0.15:1, right side of Figure 1) molar ratio in the synthesis.

Iron-based superconductors’ chemical formulas are most often given relative to the structure type to which they belong. For self-consistency within this paper, we give all chemical formulas relative to one selenium in the formula unit, regardless of structure type. To help distinguish between the many phases shown in Figure 1 and described in the text, we have appended the approximate separation between the FeSe layers in Ångströms to each formula and use these values to refer to the phases throughout the text, akin to product numbers used in molecular synthesis schemes. So, for example, phases reported elsewhere as K_0.3_(NH_3_)_*x*_Fe_2_Se_2_ and K_0.6_(NH_3_)_*x*_Fe_2_Se_2_ would be referred to here as K_0.15_(NH_3_)_*x*/2_FeSe­(7.4) or product **7.4** and K_0.3_(NH_3_)_*x*/2_FeSe­(7.9) or product **7.9**.^22^

## Results and Discussion

### Ammonia-Rich Phases

The syntheses of ammonia-rich potassium iron selenide intercalates were followed *in situ* by a similar method used to study the lithium ammonia intercalates on the beamline I12 at the Diamond Light Source, UK.^13^ As described in the Experimental Methods section, reactions were performed in the K/FeSe stoichiometric ratio of 0.3:1 and 0.15:1, which correspond to the two sides of the reported phase gap in the ammonia-poor systems.^22^

The reaction with a more potassium-rich stoichiometry of 0.3 K/FeSe proceeded smoothly from the starting material to product **9.9**, with starting material peak intensities diminishing as product peak intensities increase, appearing to give a full and near-direct conversion, as seen in Figure 2. A convenient way to follow the reaction visually is to look at the low-*Q* region, as the position of the first peak from each phase is a 00l reflection corresponding to the interlayer separation. A single, low-intensity reflection from crystalline intermediate **11.5** was observed briefly at a *Q*-value 0.53 Å^–1^ after about 20 minutes, which matches the strongest reflection of an intermediate observed in the 0.15 K/FeSe reaction described later. The final product of the reaction has a primitive tetragonal cell and good agreement with the data is achieved using a similar model to that of the ammonia-rich lithium intercalate.^13^ Remarkably, the inter-FeSe-layer separation of this ammonia-rich potassium intercalated product **9.9** is significantly smaller than the 10.6 Å separation of the ammonia-rich lithium intercalates of iron selenide, despite the larger ionic radius of potassium compared with lithium. Rietveld refinement against product **9.9**’s XRPD pattern shows that the N1 site, which we will hereafter refer to as site *A* as it is labeled in Figure 1, has a distance from the Se1 position of 3.71 Å. This distance compares well with the Se–N distance in the deuterated-amine-rich lithium intercalate of FeSe (3.710 Å, Se–D–N hydrogen bonding), supporting the conclusion that nitrogen does occupy this site. Hydrogen site positions and occupancies could not be determined from the X-ray data of this phase. When refining site *A* as purely nitrogen with three equivalents of hydrogen in an assumed 8-fold disordered arrangement around it at 1 Å distances, the site occupancy refines to 1.73(5). Even when discounting the H sites and using the form factor of Ne to overestimate the concentration of ammonia’s scattering power, the site freely refines to have an occupancy of 1.45(5), suggesting that the more electron-rich potassium shares this site. The K2 site, which lithium occupies in the analogous compound and will be referred to as site *B*,^13^ refines with a potassium occupancy of 0.00(2), indicating it is a vacant site, which tallies with the observation that the site *A*–site *B* distance of 2.11 Å is too short for a H_3_N–K interaction. Refining site *A* as a mixed K/N site (using the form factor of *K* + 1 for K and Ne for N) gives a ratio of 0.45(6):0.55(6) but given that the data collection methods were not optimized for structural refinement purposes, we have low confidence in the absolute value of the refined site occupancy. Instead, we assign a nominal composition K_0.3_(NH_3–*x*_)_0.7_FeSe to this product **9.9** based on the assumptions that all supplied potassium has inserted into FeSe, site *A* is fully occupied, and site *B* is fully vacant. This structure is shown in Figure 1 alongside other phases reported here. A full description of our treatment of the ammonia-rich structures is given in Discussion S1 on page 3 of the SI. The fit and structural model for Product 9.9 are given on page 4 of the SI in Figure S2 and Table S1, respectively. The necessity of potassium to share a site with ammonia due to size constraints explains why the interlayer separation of this intercalate is smaller than in the ammonia-rich Li/NH_3_ analogue(10.6) to this phase. There is no evidence of a superstructure establishing in the basal plane of the unit cell from K/NH_*x*_ long-range ordering. It is possible that local intra-layer ordering occurs, but does not propagate along the stacking axis.^23^

**Figure 2:**
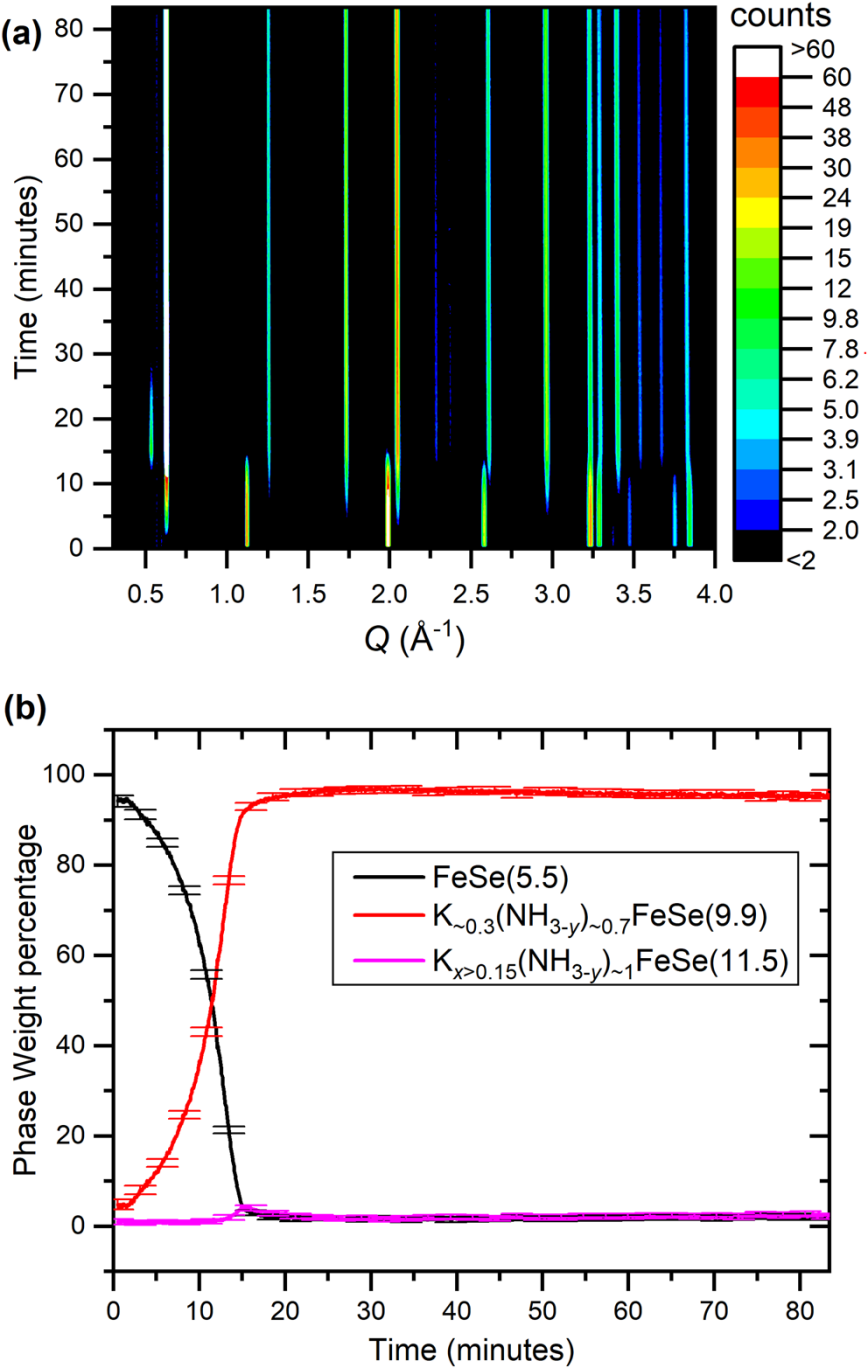
(a) Background subtracted diffraction patterns of the 0.3 K/FeSe reaction in liquid ammonia as a function of time in a contour plot. (b) Weight percentages of crystalline reactants and products in the reaction extracted from systematic Rietveld refinements against each pattern.

A more complicated story emerges in the reaction with the potassium-poor stoichiometry 0.15 K/FeSe, where the evolution of the diffraction pattern is shown as a surface plot in Figure 3a. Multiple intercalated iron selenide phases are present throughout the reaction, as can be observed by the presence of multiple peaks at low-*Q*, corresponding to phases with different *c* lattice parameters, as highlighted in Figure 3b,c. The peak at a *d*-spacing of 11.55 Å appears rapidly at the start of the reaction and then decreases in intensity. This peak belongs to a crystalline intermediate **11.5**, which is presumed to be the short-lived and low-abundance phase observed in the potassium-rich reaction described above. In the early stages of the reaction, the other well-defined low-*Q* peak has a *d*-spacing of 9.88 Å and belongs to a phase indistinguishable from that synthesized in the 0.3 K/FeSe reaction, product **9.9** described above (Table S4, page 8 of the SI). In the first 5 min the intensity of peaks from product **9.9** grow relative to intermediate **11.5** (Figure 3b), but then this balance appears to shift in favor of intermediate **11.5** again after 20 minutes, before eventually shifting back to favor the product. The initial decrease in intensity of the product **9.9** peaks occurs at the same time as a shift in position, such that when the 001 peak of the product begins to increase in intensity again its position is roughly constant at a *d*-spacing of 10.20 Å and this peak belongs to the final product **10.2** of the 0.15 K/FeSe reaction as seen in Figure 3c. We discuss the origin of this shifting equilibrium after first describing each newly observed phase.

**Figure 3:**
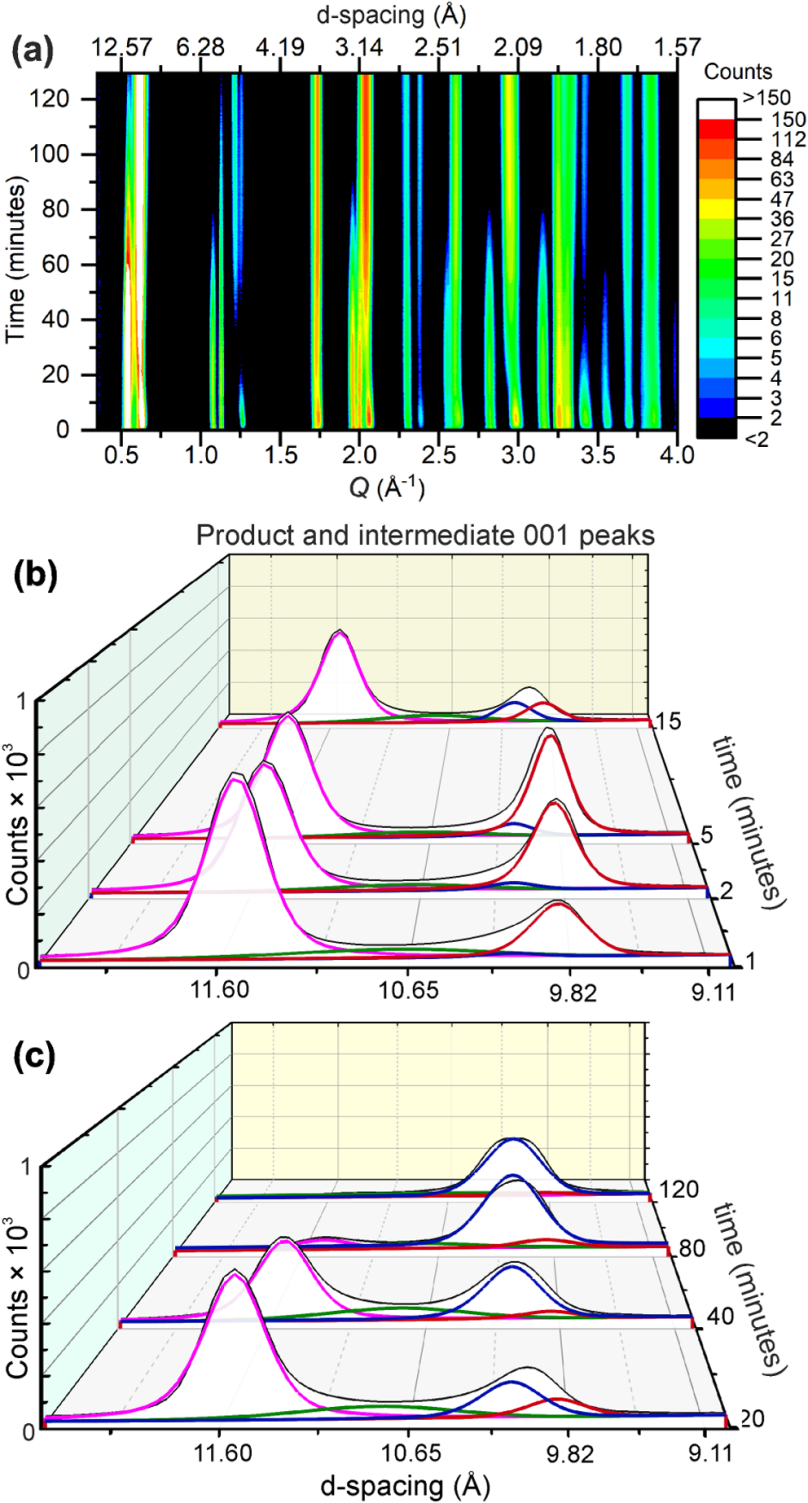
(a) Background subtracted diffraction patterns of the 0.15 K/FeSe reaction in liquid ammonia as a function of time as a contour plot. (b, c) Evolution of the product and intermediate 001 peaks (thin black line with white background), with thicker color-coded trace lines to show individual phase contributions to the data as extracted by Rietveld fitting. There is a shifting equilibrium between the crystalline intermediate **11.5** (pink) and products **9.9** (red) and **10.2** (dark blue), mediated by poorly crystalline intermediate **10.8** (green).

Product **10.2** is again a new ammonia-rich phase with an interlayer separation larger than that observed in product **9.9**, but still below that of the ammonia-rich lithium phase at 10.6 Å and far below that of intermediate **11.5**. Refining site *A* as fully occupied by a combination of N and K gives product **10.2** a more nitrogen-rich K/N ratio than product **9.9**, with refined values of 0.30(4):0.70(4). The occupancy of potassium on site *B* in product **10.2** again refines to 0.00(2). This shows that site *A* is shared between potassium and the nitrogen of ammonia once again, and the main difference to product **9.9** is a higher content of ammonia relative to potassium on site *A*, consistent with the smaller K/FeSe ratio in this reaction. We assign product **10.2** here to have the nominal composition K_0.15_(NH_3–*x*_)_0.85_FeSe­(10.2), based on the same assumptions made for product **9.9**’s composition.

Refined site occupancies for intermediate **11.5** show that site *B* has a 0.16(3) partial occupancy of potassium and site *A* has a significantly lower ratio of potassium/nitrogen (K 0.16:N 0.84(5)) relative to that of the products. These trends in occupancies suggest the identity of intermediate **11.5** is an ammonia-rich potassium iron selenide phase in which K solely occupies site *B* and NH_3_ site *A*, analoguous to the reported ammonia-rich lithium intercalate of iron selenide.^13^ Indeed, intermediate **11.5** has an inter-FeSe-layer separation that is consistent with replacing lithium with potassium in that reported structure, based on the ionic radii. We assign intermediate **11.5** a nominal composition K_*x*_(NH_3–*y*_)­FeSe­(11.5) (0.15 < *x* < 0.3, *y* > 0).

There lies a region of broadening between the low-*Q* peaks of the products and intermediate **11.5** that cannot be explained by conventional symmetric peak shapes. Broadening of this nature indicates that the conversion from intermediate **11.5** to products **9.9** and **10.2** occurs through some intermediate, highly metastable structure: a poorly crystalline intermediate **10.8**. The structure of intermediate **10.8** is expected to be partway between intermediate **11.5** and the products. We include this as a separate phase in our modeling with lattice parameters restrained to be partway between the products and intermediate **11.5**, and constraints on the atomic occupancies and thermal displacement parameters to be an approximate average of these three phases. This can be seen in Figure 3b,c as the green peak. Intermediate **10.8** has only one significant Bragg peak contribution to the diffraction pattern, which occurs in the 0.5–0.7 Å^–1^ *Q* region. Higher-*Q* Bragg reflections appear broadened into the background, which the broad peak shape of our model accounts for.

A question arises as to why do the two systems proceed via such different reaction pathways? When the solution is potassium-poor in the ratio 0.15 K/FeSe, intermediate **11.5** forms before either of the products as shown in Figure 4a,b, suggesting it is a precursor to their formation. The initially formed product **9.9** is richer in potassium than final product **10.2**, and is indistinguishable from the product **9.9** formed in the more potassium-rich solution, 0.3 K/FeSe. This implies that product **9.9** is a kinetic product that competes with the formation of the final thermodynamic product **10.2**. This is further evidenced by observing the FeSe phase percentage as a function of time in Figure 4a, which drops rapidly in the first few minutes but then plateaus to around 18 wt % after 10 min: indicating that all the potassium has been used to consume only part of the starting material. To accomplish conversion between the two products, the balances of K and NH_3_/NH_2_ in the layers must be adjusted. During this conversion process, the phase percentage of product **9.9** begins to decrease and the percentages of the intermediates **11.5** and **10.8** begin to increase once again. This fluctuation of the phase percentages shows that the intermediates mediate the conversion from K_0.3_(NH_3–*x*_)_0.7_FeSe­(9.9) to K_0.15_(NH_3–*x*′_)_0.85_FeSe­(10.2), and that the formation of product **9.9** from **11.5** is reversible. We propose that the ammonia displaces potassium from site *A* in product **9.9** and drives it to site *B* to form intermediate **11.5** before both are ejected from the structure to form what becomes the final product of the potassium-poor reaction **10.2**. After 25 minutes, when K_0.15_(NH_3–*x*_)_0.85_FeSe­(10.2) is established as the product phase, the phase percentages of intermediates **10.8** and **11.5** decrease as the percentage of product **10.2** increases. An overview of this reaction scheme is given in Figure 4c, the observed phase percentages as a function of time can be explained by *k*_1_ > *k*_2_ and *k*_–2_ < *k*_2_ > *k*_3_. The transformation from intermediate **11.5** to product **10.2** is labeled as irreversible under these chemical conditions due to the potassium extruded in this step being consumed by the remaining FeSe starting material, but we might expect the reverse process to be achievable by the addition of potassium to the solution in the absence of unreacted FeSe. This hypothesis cannot be tested without further nontrivial experiments on powder precipitates while they are stored in liquid ammonia solution.

**Figure 4:**
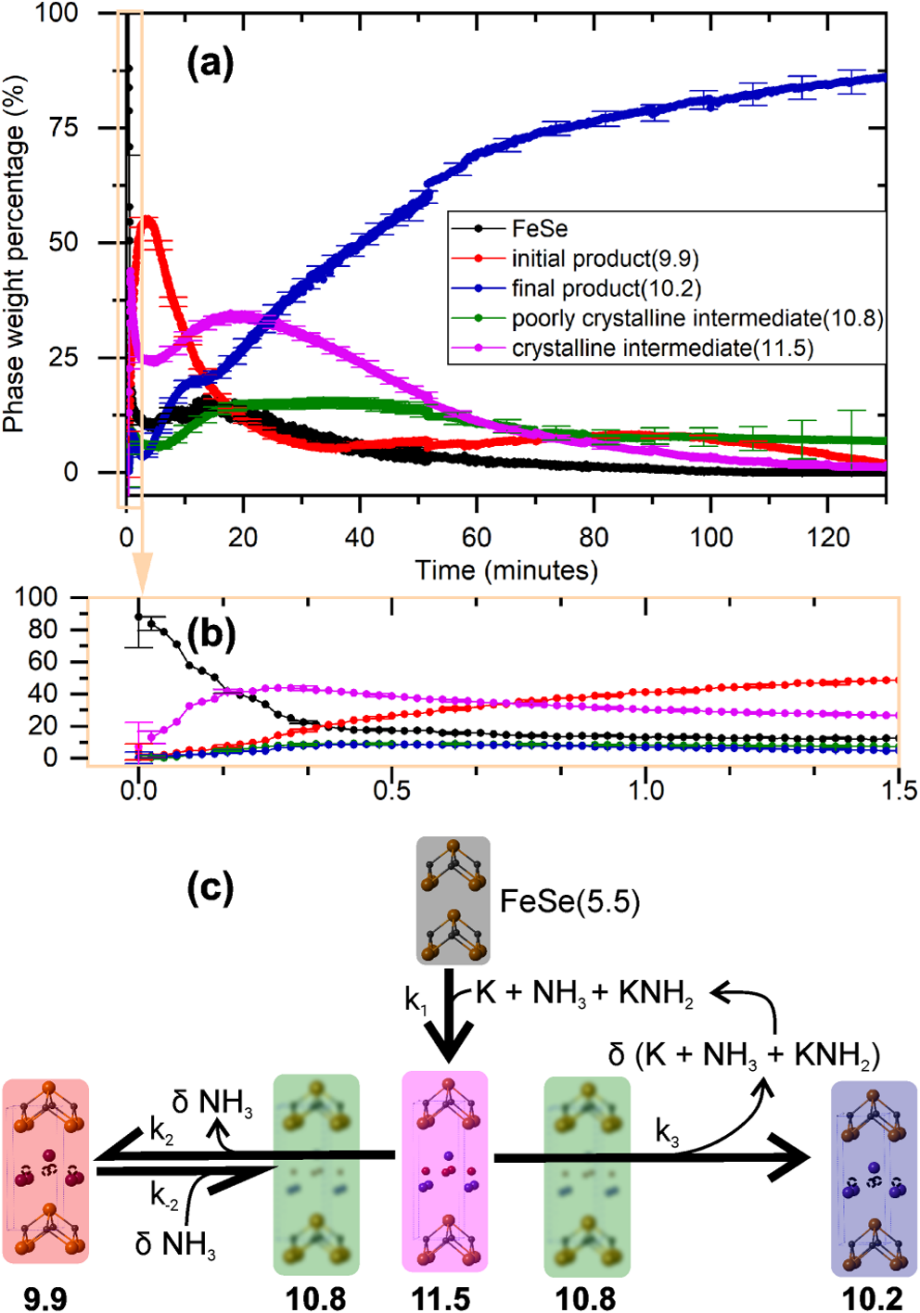
(a) Weight percentages of crystalline reactants and products in the 0.15 K + FeSe reaction in liquid ammonia as extracted from the Rietveld refinements with (b) showing the same data in the first 90 s. (c) Proposed reaction scheme.

We note that it is not possible to definitively say, from the data collected, whether products **9.9** and **10.2** form as two separate phases with a phase gap between them as we have assumed above or whether the initially formed product **9.9** converts to the final product **10.2** as a single phase that undergoes a continuous expansion in lattice parameters via a smooth change in composition. The justification for our choice to present these as separate phases and the alternative interpretation of these being a single phase of varying composition can be found in the SI (Discussion S2, pages 9 and 10).

### Ammonia-Poor Phases

In the normal procedure of handling the ammonia-rich potassium intercalates of iron selenide in the laboratory, a partial loss of ammonia occurs on drying the sample under vacuum to leave the ammonia-poor phases, which is why *in situ* diffraction while the solids are suspended in liquid ammonia is so important. Reammoniation of deuterated ammonia-poor samples back to the ammonia-rich samples was attempted as described in the Experimental Methods section; however, no reabsorption was observed. For this reason, no further characterization of the structure and properties of the ammonia-rich phases and intermediates using neutron diffraction could be performed, and we now discuss the ammonia-poor variants of these intercalated iron selenides that were first reported by Ying et al.^22^

Potassium-ammonia-poor iron selenide samples of stoichiometry 0.15 K/FeSe and 0.3 K/FeSe were synthesized *ex situ* to form products **7.9** and **7.4**, respectively, as described in the Experimental Methods section. The superconducting transition temperatures of the samples were 41 and 30 K respectively, consistent with the work by Ying et al.^22^ XRPD data were collected for both ammonia-poor products using the I11 beamline at the Diamond Light Source, UK. PND data were collected on deuterated samples using the GEM diffractometer at the ISIS neutron source, UK. Rietveld refinements against the synchrotron XRPD are complicated by significant asymmetric peak broadening. Modeling the data with a single phase produced adequate quality fits, but refinements of this nature contain unconvincing thermal displacement parameters and site occupancies. The degree of asymmetric broadening in the peaks of both ammonia-poor phases appears to vary from sample to sample, indicating some degree of nonuniform phase width and phase separation in the samples. Modeling of product **7.4** can be achieved with multiple phases each constrained to have the same atomic coordinates and occupancies but with slightly different unit cell parameters and different peak shapes. The greater the number of phases, the further the agreement factor can be lowered (see Figures S15–S17, pages 21–23 of the SI). Adding phases in this manner produces an improvement to the fit but it is misleading to think of these as truly distinct from one another, and no reliable information about their chemical differences can be extracted.

Chemical intercalations performed rapidly at low temperatures (under kinetic control) can often lead to inhomogeneous samples. The resulting powder diffraction patterns sometimes exhibit unusual broadening, which may be the result of multiple overlapping well-defined phases (each having a unique composition within the crystallites) or a phase separation within the crystallites themselves, leaving domains of varying composition. Examples of the latter are more prevalent when multiple phases of similar structure and composition can be formed, such as in the deintercalation of LiNiB.^24^ The broadening in the synchrotron XRPD observed in the ammonia-poor products is indicative of inhomogeneity in the amount of the intercalated K^+^, NH_3_, and NH_2_^–^ species between the layers, but the smooth curvature on the Bragg peaks and lack of additional maxima around the 002 reflection imply that there are no other well-defined interlayer separations that repeat over a long enough range to constitute being treated as a separate phase. This suggests that the variation in the interlayer separation probably occurs within the crystallites of these product phases.

We chose to fit the diffraction pattern of product **7.4** with a stacking fault supercell approach to determine this distribution of interlayer separations. A unit cell containing 100 FeSe and 100 K/N layers (∼3.4 × 3.4 × 740 Å) was constructed, and the position of each of the 100 FeSe layers was refined along the stacking axis of the supercell. Similar supercell approaches have been successfully employed by the authors to extract useful information from the diffraction patterns of partially disordered layered materials.^25,26^ The positions of the K/N layers were fixed to be centered between the shifting FeSe layers. The result is shown in Figure 5a–c, and produces sensible values for the distribution of FeSe interlayer separations (Figure 5d) with a large fraction between 7 and 8 Å, weighted more heavily towards 7 Å. The 7 and 8 Å extremes represent sensible interlayer separations that might be found at the limiting cases of an interlayer separated entirely by potassium or entirely by ammonia, respectively (based on the structures of K_0.4+*x*_Fe_0.8–*y*_Se­(6.9) and Li_0.6_(NH_2_)_0.2_(NH_3_)_0.4_FeSe­(8.2)).^12,27^ Observing how the interlayer separation of each layer in the model differs from that of its neighbors reveals that layers of similar interlayer separation are more likely to neighbor each other than layers of very different interlayer separation, as shown in Figure 5e.

**Figure 5:**
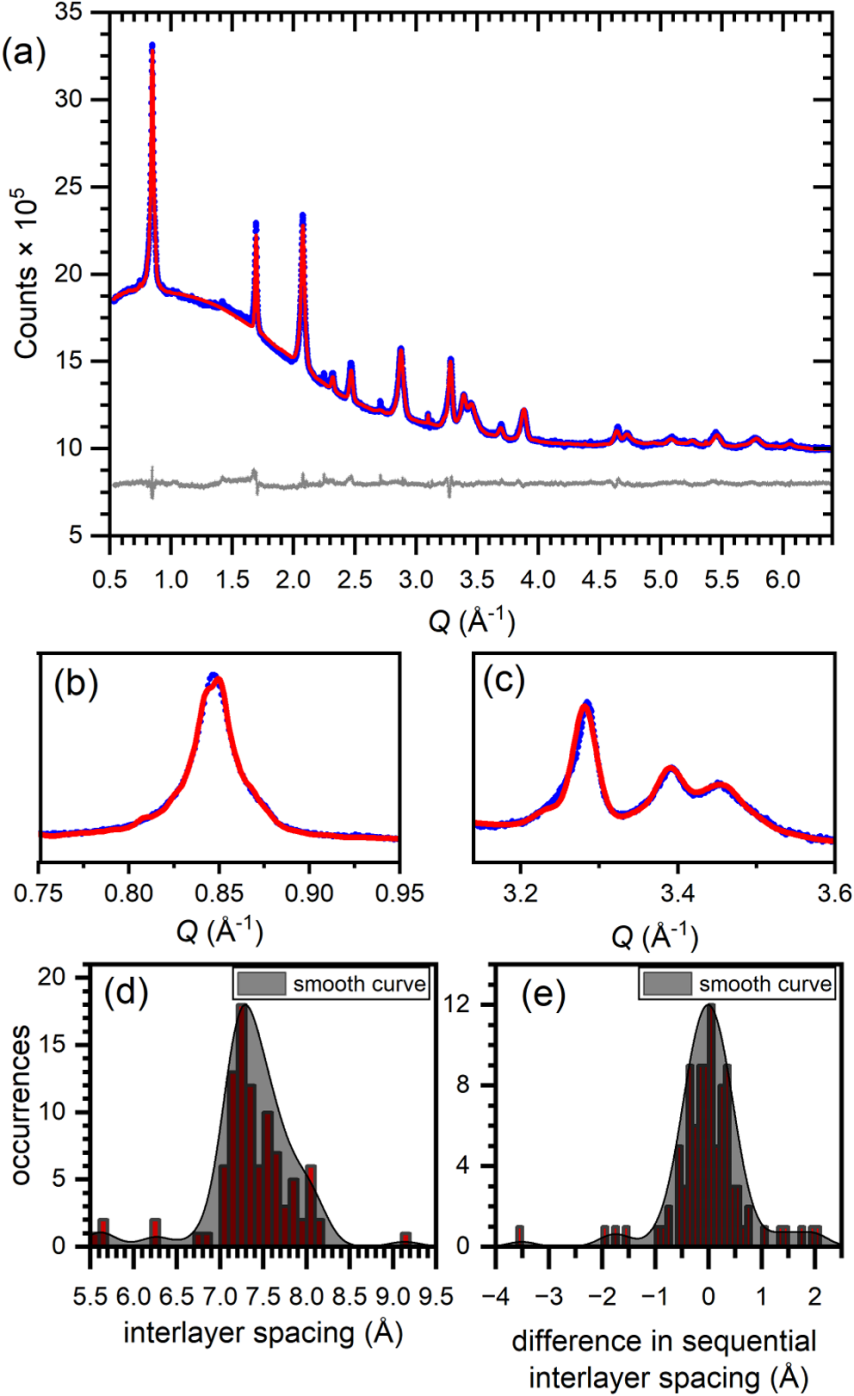
(a) Rietveld refinement against XRPD for product **7.4** using a 200-layer supercell of FeSe and K/N layers. (b, c) Select zoomed regions of this refinement. (d) Histogram plot of the distribution of interlayer separations in the refinement result. (e) Histogram plot of the difference between sequential interlayer separations in the refinement result.

Product **7.9**, which is the product of the 0.15 K/FeSe reaction once isolated in a dry inert atmosphere, may be modeled in a similar fashion to product **7.4** as shown in Figure 6. As above, a three-phase model with tetragonal anisotropic peak broadening is required to provide an adequate fit to the peak shapes, but it may also be described with a variable interlayer separation supercell model. Free refinement of the interlayer separations gives a large maximum just below 8 Å with a broad set of less frequent values around it that is more greatly weighted toward lower interlayer separations down to 7 Å. Layers of similar interlayer separation are also grouped together in this sample.

**Figure 6:**
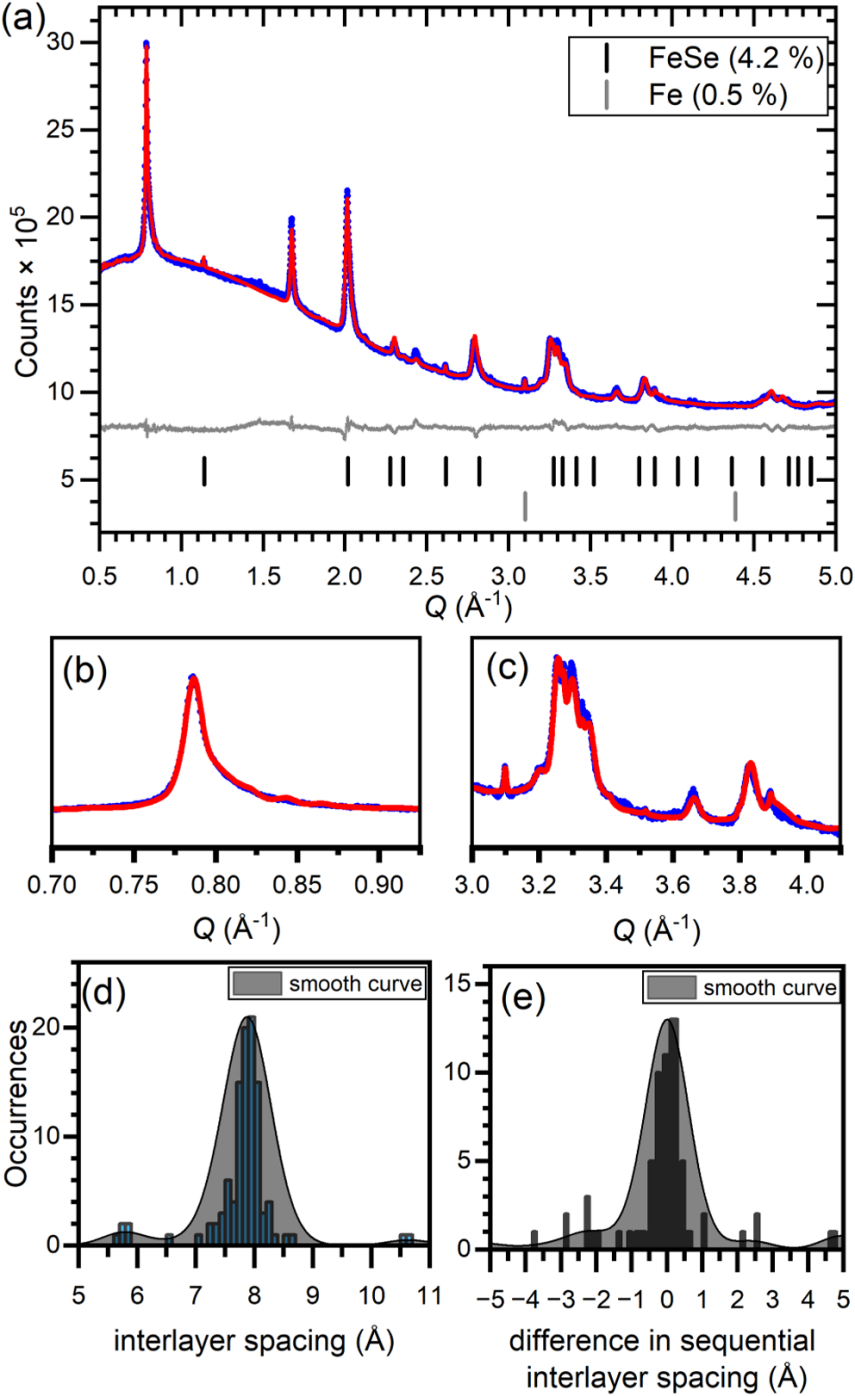
(a) Rietveld refinement against XRPD for product **7.9** using a 220-layer supercell of FeSe and K/N layers. (b, c) Select zoomed regions of this refinement. (d) Histogram plot of the distribution of interlayer separations in the refinement result. (e) Histogram plot of the difference between sequential interlayer separations in the refinement result.

Due to lower resolution of the NPD relative to the synchrotron XRPD, it was decided to model the products as single phases using Stephens-type anisotropic peak broadening adapted for time-of-flight data so as to give a picture of the average compositions.^28^ This broadening model gave similar agreement factors to using three phases (*R*_wp_ 4.75 and 4.85%, respectively for product **7.4**). Choosing to refine with a single phase allows us to use the NPD to describe the average structure and occupancies while using the XRPD to describe the inhomogeneity. Rietveld refinements against both data sets suffered large correlations between the occupancies of the potassium, nitrogen, iron, and deuterium sites; meaning there was little change in the agreement factor between models with sensible stoichiometries and models with implausible N/D ratios. In light of this, it was necessary to fix the potassium content in both models to that supplied by the reaction stoichiometry. Sensible N/D ratios could then be refined, but it should be noted that these constraints could bias the refined stoichiometries.

Rietveld refinement against NPD data for the deuterated product **7.4** is shown in Figure 7a with the model reported in Table S6 on page 14 of the SI. The structural trait of potassium and ammonia sharing a crystallographic site that we observe in the ammonia-rich intercalate structures is continued here. In this average structure, neither ammonia nor potassium have their ideal bond distances fully satisfied by sharing the average site; the Se–D distance of 2.50(5) Å is shorter than usual for a Se–H bond in other intercalates (∼2.75 Å), and the K–Se distance of 3.454(2) Å is slightly longer than that in K_2_Se (∼3.33 Å). This mismatch will, in part, give rise to local variation in the structure, as the environment about ND_3_ and K^+^ will locally distort to optimize the distances, which explains the highly anisotropic shape of selenium’s thermal displacement ellipsoid: elongated along the *c* axis. The refined site occupancies indicate that product **7.4** has a stoichiometry of K_0.3_(ND_2.6(4)_)_0.14(2)_FeSe, which suggests a mean iron oxidation state of Fe^1.76(6)+^.

**Figure 7:**
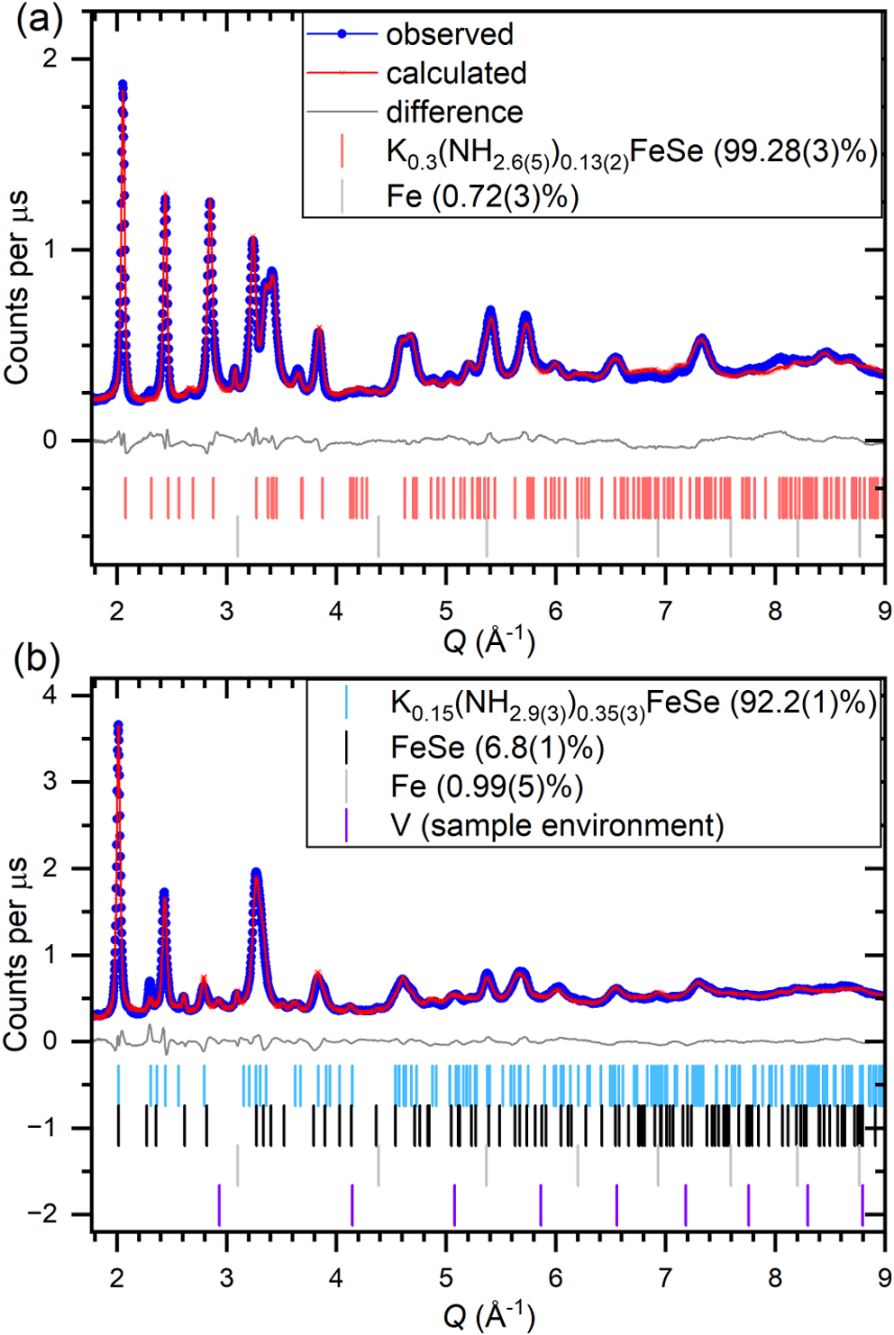
Rietveld against neutron powder diffraction from Bank 4­(2θ = 64°) of the GEM diffractometer for (a) K_0.3_(ND_2.6_)_0.14_FeSe­(7.4) and (b) K_0.15_(ND_2.9(3)_)_0.35(3)_FeSe­(7.9). Fits to the data of other detector banks are given in Figure S10 on page 13 of the SI.

A broad peak, not observed in the XRPD and likely to originate from magnetic ordering, is observed at room temperature in the low angle detector banks 1 and 2 at a *d*-spacing of around 10.8 Å, but no further distinct peaks of magnetic origin are observed. This reflection was indexed to a 2√2 expansion of the *a* cell parameter, and can be refined as an antiferromagnetic ordering with an ordered moment on Fe of 1.12(5) μB as detailed in Discussion S3 on page 15 of the SI. This moment size is indicative of some degree of phase separation into antiferromagnetically ordered and superconducting phases that are not distinguishable by NPD, as appears in antiferromagnetically ordered K_0.8_Fe_1.6_Se_2_, which has *T*_N_ ≈ 559 K.^29^ Such a phase separation is consistent with our analysis of the XRPD and the low (10%) superconducting volume fraction of the sample, shown by SQUID magnetometry in Figure S12 on page 18 of the SI.

Rietveld refinement against PND data for product **7.9** is shown in Figure 7a. No indication of magnetic ordering was observed for this sample. The refined nuclear model gives a product stoichiometry of K_0.15_(ND_2.9(3)_)_0.35(3)_FeSe­(7.9) giving an average iron oxidation state of Fe^1.89(11)+^. The larger interlayer separation of product **7.9** relative to the more potassium-rich product **7.4** is partially explained by the interlayer site refining as fully occupied in the former but containing 12% vacancies in the latter. But the different sizes of the site-sharing potassium and ammonia also contributes to this larger interlayer separation, since less potassium leaves room for more ammonia, which tends to have longer N–H–Se distances (3.5-4) than typical K–Se distances (3.3-3.5).^30,31^ The refined Se–D distance of 2.76(1) Å compares well with the Se–D bond reported in the ammonia-poor lithium intercalate (∼2.75 Å),^12^ but this now makes the 2a site to Se distance 3.72 Å: very unfavorable for a K–Se bond. A site displaced from nitrogen by around 0.6 Å in the *c* axis was identified in the neutron diffraction, which consistently refined with nonzero scattering length. This distance is too close to the nitrogen site to be deuterium but is 3.32(2) Å from selenium, which is the right distance to correspond to a K–Se bond. The close approach of potassium on this 4e Wyckoff site and nitrogen on the 2a Wyckoff site forbids them from being occupied at the same time in the local structure. Likewise, the 4e site of potassium could only be a maximum of half occupied since the two positions are only 1.26 Å, prohibiting simultaneous occupation. This split-site model for K^+^ could alternatively be pictured as potassium sharing the 2a site with nitrogen, with an hourglass shaped displacement ellipsoid pointed towards the nearest selenium sites.

XANES measurements on nondeuterated samples of products **7.9** and **7.4** confirm that there is a shift to lower energies indicating a reduction in the Fe *K*-edge relative to the FeSe parent material for both phases, which is greater for the more potassium-rich product **7.4**. The edge positions as judged by the zero-crossing of the second derivative are 7118.60, 7118.31, and 7118.11 eV for parent phase FeSe, K_0.15_(NH_∼2.9_)_∼0.35_FeSe­(7.9), and K_0.3_(NH_∼2.6_)_∼0.14_FeSe­(7.4), respectively. It was not possible to establish the absolute values of the oxidation states from the edge position shift by calibration against a known system. An attempt to use LiFe_*x*_(OH)_1–*x*_Fe_1–*y*_Se as a reference system overestimates the amount of reduction here beyond what could be chemically reasonable (Fe^1.73+^ and Fe^1.55+^, respectively),^18^ presumably due to differences in the local environment between the two systems. The oxidation states assigned by Rietveld refinement against the NPD are consistent with there still being a linear relationship between the oxidation state and shift in edge position for the potassium and ammonia-intercalated iron selenides, with an edge shift of one electron volt corresponding to an approximate 0.44 change in oxidation state in this system. A plot of oxidation state derived from neutron diffraction versus edge position is given in Figure S14 on page 20 of the SI.

### Ammonia-Free Phases

Ying et al. observed that a superconducting phase containing only potassium between the layers can be isolated by heating product **7.9** at 200 °C. Our thermogravimetric analysis (shown in Figure S13 on page 19 of the SI) shows a mass loss event between 200 and 300 °C in both products **7.4** and **7.9**. The sizes of the mass loss events are consistent with the mass of ammonia in each structure as modeled in refinements against the neutron data (1.56% for product **7.4** and 4.04% for product **7.9**). The decomposition processes on warming the two products from room temperature to 400 °C have been measured *in situ* by XRPD on beamline I11. A useful range of the data sets can be seen in Figure 8. In each case, the lowest *Q* (002) reflection of the starting phase moves to a slightly lower *Q* from 25 to 200 °C as thermal expansion occurs, but at around 200 °C the peak begins to decrease in intensity and a second peak begins to grow in at higher *Q*, consistent with a product phase where ammonia has been lost. The ammonia-free product peak gradually replaces the ammonia-poor product peak, accompanied by the appearance of elemental iron and iron selenide in the sample, as observed in Figure 8 by the growth of the FeSe 001 and Fe 110 reflections. The final product formed in both reactions is an iron vacancy containing the K_0.5–*x*_Fe_1–*y*_Se­(7.1) phase.

**Figure 8:**
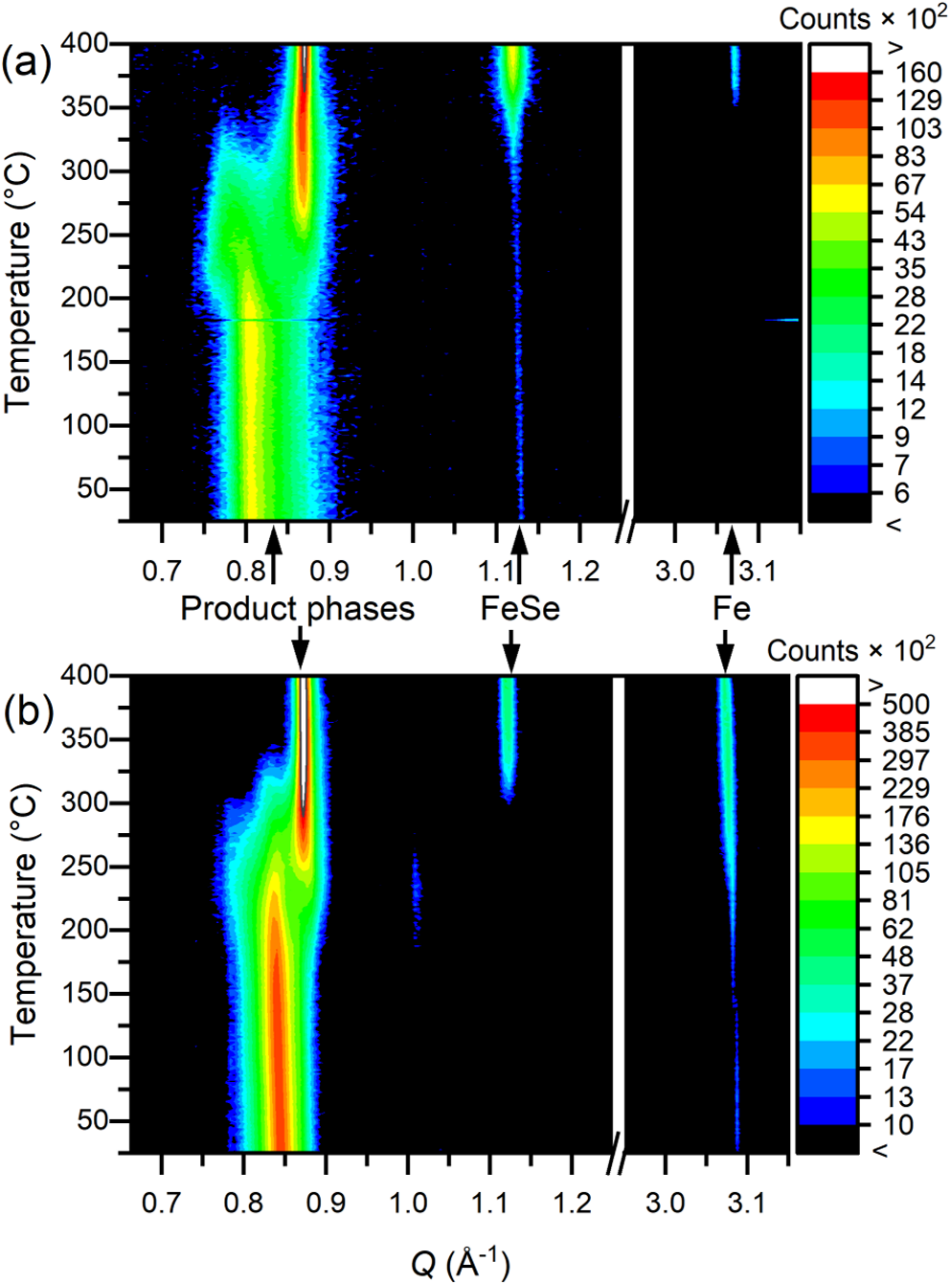
Background subtracted variable temperature powder X-ray diffraction for the thermal decomposition of (a) potassium-poor product **7.9** and (b) potassium-rich product **7.4**. the full measurement range is given in Figure S18 on page 24 of the SI.

Systematic Rietveld refinement was carried out against each of the diffraction patterns for both reactions. Phase fractions as a function of temperature are shown in Figure 9a,b, which we use to characterize the successive reaction processes occurring as a function of temperature. The decomposition of product **7.4** can be separated into stepwise processes as approximated in Figure 9c as steps **1**, **2**, and **3**. The loss of ammonia from the interlayer and change of the main phase from K_0.3_(NH_∼2.6_)_∼0.14_FeSe­(7.4) to K_0.5–*x*_Fe_1–*y*_Se­(7.1) perfectly coincides with the appearance of the 110 reflection of iron, indicating that iron is expelled from the FeSe layers at the same time as ammonia is lost/amide is decomposed, following step **1**. We will refer to the phase formed as product **7.1** but this is not a line phase, it has variable values of 0 < *x* < 0.2 and 0 < *y* < 0.3. As the temperature further increases, we see a phase separation within the product to release binary Fe_1–*y*′_Se by step **2** which is presumably driven by the favorability of consolidating potassium into the vacancies left by ammonia and amide. As this process occurs, the now-more-K-rich product **7.1** ejects more elemental iron to prevent over-reduction of the Fe in its Fe_1–*y*_Se layers. The amount of elemental iron in the diffraction pattern first plateaus and then decreases slightly above 330 °C, which indicates that the expelled elemental iron is redistributed and reabsorbed either by Fe_1–*y*′_Se or by the product **7.1** following step **3**. Fe is most likely to be re-entering the Fe_1–*y*′_Se phase but it was not possible to reliably determine the value of *y*′ in the Rietveld refinements. It is clear that the expulsion of iron from the sample happens at a faster rate than the redistribution of iron to other parts of the sample, leading to metastable iron-deficient Fe_1–*y*′_Se or K_0.5–*x*_Fe_1–*y*_Se compositions.

**Figure 9:**
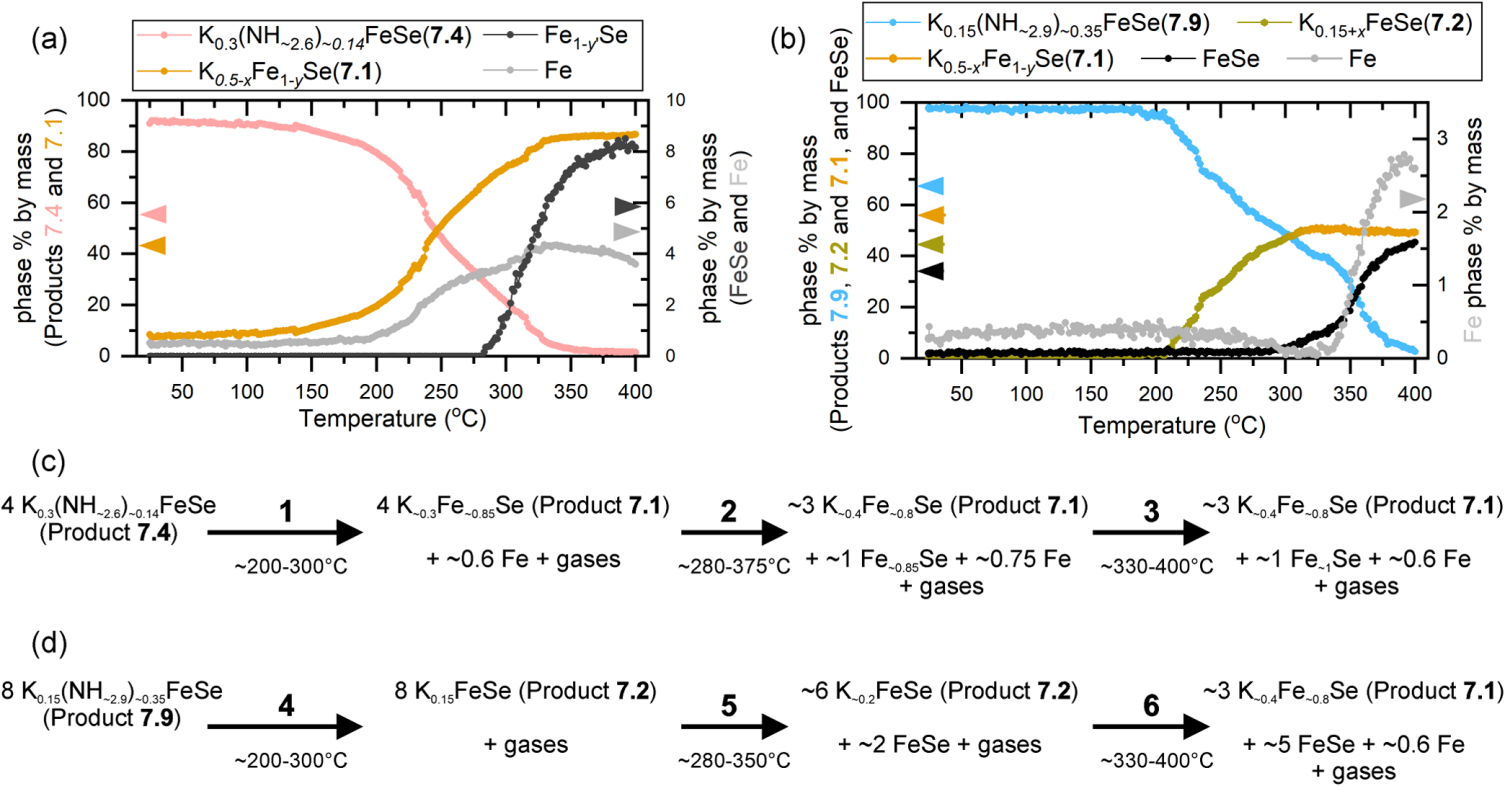
Phase fractions of the reactant and products in the thermal decomposition of (a) product **7.4** to product **7.1** and FeSe, and (b) product **7.9** to product **7.1** and FeSe via product **7.2**. (c, d) Simplified chemical equations for the reaction processes occurring in each step of the decomposition are provided for (a, b), respectively. The compositions given in these chemical equations after each step of the decomposition are nominal since these are continual changes rather than transitions between line phases. The balancing of the equations is also nominal, since these will depend on the exact vacancy concentrations. See the description in the article for further explanation.

The decomposition of product **7.9** also results in the formation of product **7.1**, via a stepwise process in steps **4**, **5**, and **6** approximated in Figure 9d. A crucial difference in this reaction is that the product phase begins to form before the appearance of either FeSe or Fe. Over the temperature range 200–280 °C the reaction follows step **4**, a simple expulsion of gases from the interlayer. Whereas the decomposition of product **7.4** saw elemental Fe appear coincident with this step, this time the reduction of Fe in the intercalate is not so high as to promote disproportionation and it seems a metastable K_0.15+*x*_FeSe­(7.2) phase can be formed, consistent with the study by Ying et al. who isolated this phase by annealing under vacuum at 200 °C.^22^ This product **7.2** has a very similar structure and interlayer separation to product **7.1**, but it is distinct due to the lack of vacancies on the iron site. Product **7.2** begins an internal phase separation in step **5** with the expulsion of FeSe and consolidation of K to a more-K-rich product **7.2** from 280 °C onwards. As potassium consolidates between FeSe layers it over-reduces the iron and causes a disproportionation to form the final product **7.1** and expel elemental iron in step **6** from 330 °C onwards.

## Concluding Remarks

This report has examined seven phases in the potassium- and ammonia-intercalated iron selenide phase diagram and detailed the reaction pathways between them. Structural information for these phases can be found in Table 1 and the corresponding tables in the SI. While each one is distinct, our understanding of the individual phases can be enhanced by examining them as a collective and contextualizing them in the wider iron selenide superconductor family.

**Table 1:** Summary of the Key Crystal Structure Information for the Various Products Reported Here and Locations of the Full Structural Models Given in the Supporting Information

identifier	composition	space group	*a* (Å)	*c* (Å)	temp. (K)	SI Table
11.5	K_∼0.3_(NH_3–*y*_)FeSe	*P*4/*nmm*	3.8141(6)	11.5557(7)	≈255	S5
10.2	K_0.15_(NH_3–*x*_)_0.85_FeSe	*P*4/*nmm*	3.8601(4)	10.1899(9)	298	S2
9.9	K_0.3_(NH_3–*x*_)_0.7_FeSe	*P*4/*nmm*	3.8409(3)	9.8800(6)	298	S1
7.9	K_0.15_(NH_∼2.9_)_∼0.35_FeSe	*I*4/*mmm*	3.8503(3)	15.938(3)	298	S7
7.4	K_0.3_(NH_∼2.6_)_∼0.14_FeSe	*I*4/*mmm*	3.8429(4)	14.689(2)	298	S6
7.2	K_0.15+*x*_FeSe	*I*4/*mmm*	3.8879(4)	14.407(3)	528	S10
7.1	K_0.5–*x*_Fe_1–*y*_Se	*I*4/*m*	8.6712(3)	14.173(1)	298	S8 and S9

Much of the behavior of the K-NH_3_–FeSe system can be explained by contrasting it to the Li-NH_3_–FeSe system. The larger radius of K^+^ than Li^+^ makes the former less capable of filling smaller and lower coordination sites between the layers, and more prone to sharing the NH_3_ site. While the ammonia-rich phases of K-NH_3_–FeSe do form in the synthesis, unlike the reported lithium analogues, they could not be remade by exposing the ammonia-poor phases to 1 bar of gaseous ammonia at −10 °C, indicating a higher barrier to the phase change. This is likely the result of NH_3_ being unable to stably coordinate the K^+^ within the layers in the same way it does for the more charge-dense and strongly solvated Li^+^. Such coordination where NH_3_ and K^+^ are still closely bound in the interlayer has been observed in a metastable crystalline intermediate **11.5** but is short-lived in the reaction. This intermediate is a precursor to the products, which we propose is the result of ammonia and potassium entering between the iron selenide layers complexed together before the favorable bonding of NH_3_ and K^+^ individually to the selenide layers, reduces the bonding between them, and separates them. We observe that the potassium-rich conditions form their product (**9.9**) faster than the potassium-poor conditions, and the more-K-rich product **9.9** occurs as a kinetic product in the formation of the potassium-poor product **10.2**. We observe that the conversion between product **9.9** and **10.2** occurs via the intermediate **11.5**.

The tendency for K^+^ and NH_3_ to share an average crystallographic site is continued in the ammonia-poor cointercalates. We observe that the two formally share a crystallographic site coordinated by a square prism of selenide ions when the interlayer separation is 7.4 Å. However, K^+^ is smaller than NH_3_, and when the interlayer separation is larger (7.9 Å), the K^+^ has an average displacement away from the center of the square prism in which NH_3_ resides. Presumably this separation distance is favorable for ammonia but has a considerable energy penalty to the K–Se bonding. We propose the established phase gap in the ammonia-poor phases between these two interlayer separations may occur because K^+^ cannot maintain bonding to both neighboring selenide layers at separations above 7.4 Å, but displacing K^+^ from the center of the square prismatic site is too steep an energy penalty before the system is sufficiently potassium-poor. Remarkably the phase gap is also present in the ammonia-rich phases, and the same ratios of K/FeSe of 0.15:1 and 0.3:1 seem to present its two sides, although further *in situ* synthesis experiments would be required to fully establish the exact compositional range of this gap and the phases on either side of it.

Unlike Li^+^, K^+^ is large enough to form stable K_*x*_FeSe phases without the coincorporation of NH_3_, which makes the thermal decomposition to such phases possible. We have shown that the decomposition follows the established rules for intercalated iron selenides: that a small level of reduction of Fe^2+^ can be tolerated but too much leads to disproportionation to Fe^2+^ and Fe(0). We can define the term *K*_max_ as the maximum level of potassium that can be supported between iron selenide layers in the absence of ammonia and amide before there is a disproportionation. For 0.3 K/FeSe *K*_max_ is surpassed immediately but with 0.15 K/FeSe this initially falls below *K*_max_ and it is possible to form metastable superconducting K_0.15+*x*_FeSe phases in a narrow temperature window before this succumbs to a phase separation that consolidates the K^+^ in some layers, which causes the *K*_max_ limit to be reached. We can therefore confidently assign 0.15 < *K*_max_ < 0.3, and we can approximate its value as *K*_max_ ≈ 0.2 by observing that the molar ratio of K_0.15+*x*_FeSe with respect to FeSe reaches 75:25 before any iron is extruded, meaning that a *K*_max_ containing composition has the formula K_0.15_(FeSe)_0.75_ ≡ K_0.2_FeSe.

Accurate measurement and characterization of iron-based superconductors is essential for our understanding of their physical properties, and *in situ* measurements are key to understanding the energy landscape that separates the stable and isolable compositions. The study presented here offers high-quality phase characterization and (what is currently) a rare insight into the phase transformation kinetics of a solid + solution and a solid → solid + gas reaction, elucidating multiple steps in each reaction which result from an intricate combination of competing energy barriers and thermodynamic minima. Our understanding of reaction kinetics in the solid state lags far behind that of molecular reactions, but the field is consistently growing.^32–34^ Technological developments at central facilities and for in-house diffractometers make studies into reaction processes increasingly accessible and commonplace, making further work to study phases that are stable only in solution and extract kinetic parameters from reactions such as those presented here a possibility.^35–37^ By studying processes such as these, we will be able to better understand and design synthesis routes for solids.

## Experimental Methods

### Rietveld Refinements

Refinements throughout the paper were carried out using Topas Academic version 6.^38^ The supercell models used in the Rietveld refinement of the ammonia-poor data employed the stacking fault functionality of this software.^39^ The size of the supercell used was optimized for each of the two cases. If the supercell is too small, it might fail to represent the distribution of layer separations, but if the supercell is too large, it might fail to reach a global minimum; the former can be identified by the presence of rippling in the calculated pattern, while the latter can be identified by the results of repeat iterations being fundamentally different. A 200-layer supercell was used for product **7.4** while a 220 layer supercell was found optimal for product **7.9**. Refined interlayer separations in the supercell that occur outside of the main distribution are unlikely to be physically meaningful, but it was chosen not to impose restraints since the freely refined minima did not go below an interlayer separation corresponding to no intercalation of (5.5 Å in FeSe) and did not exceed the maximum interlayer separations found in the ammonia-rich phases.

Diffraction data in the main paper have been presented in *Q*-value (=2π /*d*) for ease of comparison between the data sets, but the 2θ or time-of-flight data that the models were refined against are shown in the SI.

### *In Situ* Synthesis on Beamline I12, Diamond

6.6 mg and 13.1 mg of K were placed at the bottom of two separate 18 mm o.d. and 14 mm i.d. Pyrex ampules with a side arm in an identical manner to the work of Sedlmaier et al.^13^ These ampules were custom made by a professional glass-blower to withstand internal pressure of up to 15 bar. 150 mg of FeSe were loaded into the side arm of each so as to avoid contact with the potassium metal, and the ampule was sealed with a high-performance Rotaflo Teflon valve. The bottom of the Pyrex ampule was placed in liquid nitrogen and 3–5 mL of NH_3_ were condensed onto the alkali metal using a Schlenk line. Once the required volume of NH_3_ was condensed, the ampule was sealed at the Teflon valve, and the solid NH_3_ was allowed to melt at −78 °C in a CO_2_-isopropanol bath. At beamline I12,^40^ the ampule was clamped to a remote-controlled rotation stage, such that the FeSe in the side arm could be tipped into the homogeneous K/NH_3_ solution remotely. With the solution stirring, the solution was exposed to a monochromatic 80 keV X-ray beam, and diffraction patterns were collected every second as the FeSe in the side arm was tipped into the solution. Data were collected with a Pixium image plate detector and the diffraction rings in the collected data were integrated to produce a one-dimensional pattern using the software suite Fit2D.^41^ The ampule was surrounded by a small bowl-shaped, glass vacuum Dewar cooling bath, which was ≈ −60 °C at the beginning of the reaction. The cooling bath was not supplied with additional dry ice during the reaction, therefore, warmed toward room temperature over the course of approximately 1 h. Temperature-controlled conditions were investigated but these added excessive background to the diffraction patterns and it was found that decolourization of the K/NH_3_ solution to form KNH_2_ would occur if the solution were left too long or allowed to warm too high, making extraction of kinetic parameters infeasible during the designed experiment. Attempts to probe the transition from ammonia-rich to ammonia-poor phases were limited to *ex situ* snapshots since it would not have been possible to remove the ammonia from the reaction flasks in the beamline’s experimental hutch in a safe and controlled manner. These snapshot XRPD patterns are given in Figure S23 on page 30 of the SI. **Caution**: ammonia is volatile and toxic. When at room temperature any sealed apparatus must be able to withstand the autogenous pressure generated by the solution.

### X-ray Absorption Spectroscopy

Measurements were conducted in transmission mode on beamline B18 at Diamond with the samples sequestered from air between sheets of Kapton tape and diluted with cellulose powder.^42^ All spectra were calibrated against iron foil. The data were analyzed using Athena and Artemis, part of the Demeter software package.^43^

### Synthesis of Ammonia-Poor Phases for XRPD and NPD

The precursor β-FeSe, was synthesized via a ceramic method on the 12 g scale. Powders of iron (Alfa Aesar, 99.998%) and selenium (Alfa Aesar, 99.999%) were ground together in a 1:0.98 ratio; this small excess of iron promotes the β form over the α. The mixture was sealed in an evacuated silica tube and heated to 700 °C at 2 °C min^–1^. The reaction was held at 700 °C for 24 h before being cooled to room temperature. After extracting and regrinding the powder product in the glovebox, the powder was divided into 3 g portions, and each was individually sealed in an evacuated silica ampule for a second annealing at 700 °C for 38 h, followed by a final annealing step at 400 °C for 48+ h. The final annealing temperature favors the β phase and the ampules were quenched in ice water from 400 °C to prevent conversion back to the α phase, which may occur on slow cooling.

Pieces of potassium (Alfa Aesar, 99.95%) were cut from lumps to remove the oxidized surface, inside an argon-filled, dry glovebox. The potassium pieces were combined with iron selenide powder in the desired molar ratio and placed in the bottom of a Schlenk tube. ∼20 to 50 mL of NH_3_ or ND_3_ were condensed onto the sample chilled to −78 °C using a dry ice-isopropanol cooling bath. The reactions were stirred for 2–5 h before boiling off the solvent. **Caution**: Potassium is highly reactive, and ammonia is volatile and toxic. Air and moisture exposure must be prevented throughout. Removal of the liquid ammonia by evaporation should be done slowly and carefully. The Schlenk tube was evacuated under dynamic vacuum for 3 min before returning it to the glovebox for isolation. The ammonia-poor samples were found to be nominally air-stable (at least for short periods), but all handling and measurements were performed under inert conditions to avoid potential oxidation and decomposition.

Unsuccessful attempts to re-form the ammonia-rich phases from the isolated ammonia-poor phases were performed by exposing each phase to one bar of deuterated ammonia pressure at −10 °C and sealing them in vanadium cans for neutron diffraction in the same way that was successful in reforming the ammonia-rich lithium intercalate of iron selenide.^13^

### Neutron Powder Diffraction

Measurements used the GEM diffractometer at the ISIS Facility, UK.^44^ Rietveld refinement was carried out simultaneously against time-of-flight diffraction patterns from 1 to −5 of GEM’s detector banks using a single structural model for each sample. The *d*-spacing range covered in the refinements across banks 1–5 was 21.5–0.53 Å.

### Synchrotron XRPD and *In Situ* Thermal Measurements

High-resolution synchrotron X-ray powder diffraction measurements were performed at 15 keV with beamline I11 at the Diamond Light Source, UK.^45^ Samples in 0.5 mm diameter capillaries of ∼40 mm length were positioned on the diffractometer with a Cyberstar hot-air blower directed at them, with the hot-air stream traveling perpendicular to the capillary in a ∼5 mm window. The sample was warmed at a rate of 6 °C min^–1^, diffraction patterns were collected continuously at ∼14 second intervals using the position sensitive Mythen2 detector. This would have resulted in a small pressure of the released gases building inside the capillary during the measurement, in addition to the argon pressure increasing with temperature. However, since only a portion of the sample in the X-ray beam was heated by the hot-air stream during the measurement, the increase in pressure would have been minimized.

### SQUID Magnetometry

Measurements used a Quantum Design MPMS-XL SQUID magnetometer with a measuring field of 50 Oe to characterize the superconducting state by zero-field-cooled and field-cooled susceptibility measurements. Samples were sequestered from the air in gelatin capsules.

## Supplementary Material



## Notes

The document contains the two theta or time-of-flight data that were used for the Rietveld refinements; crystallographic parameters extracted from those refinements for each presented phase (Table 1); thermogravimetric data; XANES data; and elaborated discussions on the treatment of the *in situ* synthesis data (Discussions S1 and S2) and the magnetic peak of product **7.4** (Discussion S3) (PDF)
