# Supplementary material for: Stepwise Reactions in the Potassium and Ammonia-Intercalated Iron Selenide Superconductor Phase Diagram Followed by In Situ Powder Diffraction
Source: J Am Chem Soc. 2025 May 19;147(22):18563–75. doi: 10.1021/jacs.5c00356 (PMC12147115; doi:10.1021/jacs.5c00356)
Supplement: Supplementary file 1 [file ja5c00356_si_001.pdf]

Supplementary Materials for

Stepwise reactions in the potassium and ammonia intercalated iron selenide superconductor phase diagram followed by in situ powder diffraction

Simon J. Cassidy,∗,† Daniel N. Woodruff,† Stefan J. Sedlmaier,† Jack N. Blandy,† Christina Reinhard,‡, Oxana V. Magdysyuk,‡,§ Andrew L. Goodwin,† Silvia Ramos,‖ and Simon J. Clarke∗,†

†Department of Chemistry, University of Oxford, Inorganic Chemistry Laboratory, South Parks Road, Oxford OX1 3QR, U.K.
‡Diamond Light Source Ltd, Harwell Science and Innovation Campus, Didcot, U.K.
¶The University of Manchester at Harwell, Diamond Light Source, Harwell Campus, Didcot, Oxfordshire, OX11 0DE, U.K.
§EaStCHEM, School of Chemistry, University of St Andrews, North Haugh, St Andrews KY16 9ST, U.K.
‖School of Physics and Astronomy, University of Kent, Canterbury, CT2 7NH, Kent, U.K.

E-mail: simon.cassidy@chem.ox.ac.uk; simon.clarke@chem.ox.ac.uk

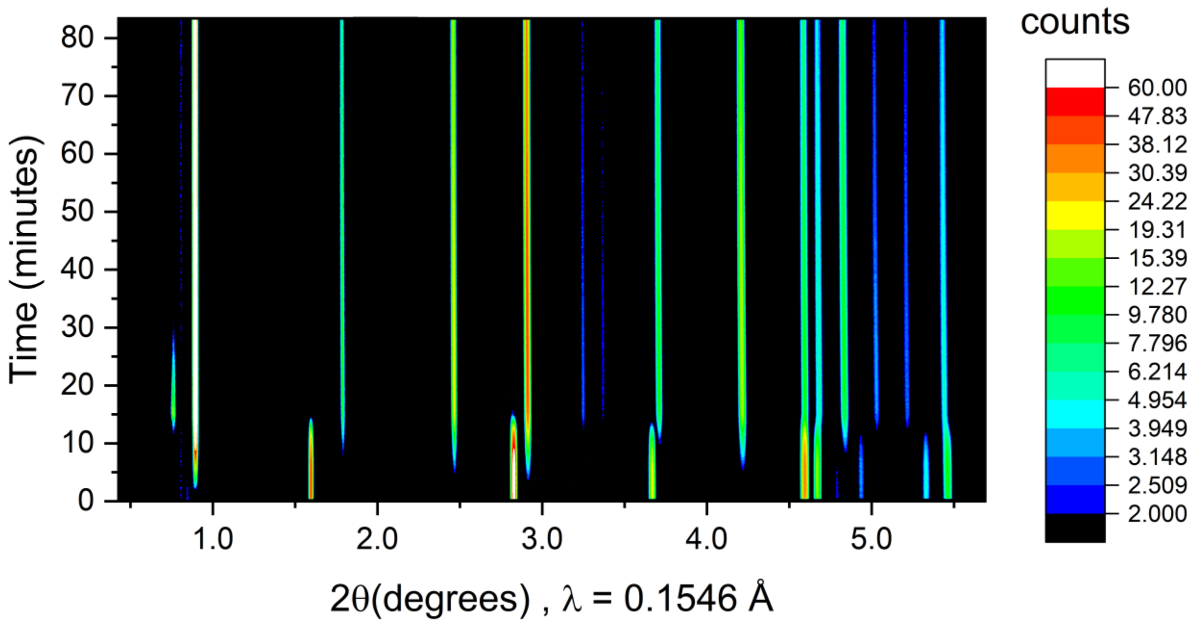

Figure S1 replot of figure 1 in two theta degrees instead of Q-value

Discussion S1: Refinement of the structures of the ammonia-rich phases. Diffraction patterns collected on beamline I12 at diamond light source during the *in situ* measurement of the synthesis of potassium and ammonia intercalated iron selenides were not well optimised for structural refinement purposes. The refined site occupancies are highly dependent on the treatment of:

(1) The thermal displacement parameters: A single isotropic thermal displacement parameter has been refined as the thermal displacement for every site. Constraint of the thermal displacement in this way is standard practice when the data quality does not allow for accurate refinement of all the thermal displacement parameters, but the true thermal displacements are likely to be anisotropic and to be much higher for the interlayer species than they are for Fe or Se. There is strong correlation between the thermal displacement parameters and the occupancies.

(2) An absorption correction: The thick walls of the glass reaction vessel, the glass Dewar, the solvent in the Dewar, the ammonia solution in the reaction vessel and a not-well-determined amount of sample will all lead to some absorption even with the 80 keV beam, which will correlate with the thermal displacement parameters and occupancies. This cannot be accurately corrected for.

(3) The form factor used to model ammonia /amide: this may be reasonably treated as N^{3-} or N with three equivalents of Hydrogen in a disordered arrangement at 1 Å distance from the N site, using the model in Sedlmaier et al., Journal of the American Chemical Society, 2014, 136, 630–633, as a basis to approximate the H positions. The refinement software did not have a pre-defined form factor for N^{3-} so O^{2-}, F^{-} or Ne offer isoelectronic options, but these will overestimate the concentration of electron density.

Although the site occupancies are individually unreliable, we use them as a comparative guide to show the relative scattering powers of what are referred to as Site *A* and Site *B* in the different ammonia-rich products and intermediates in the main text. When discussing the refinement of site *A* as a mixed K/N site to give a ratio of the two species on the site we have used the form factors of K+1 and Ne for each case, but the choice is somewhat arbitrary as the given refined values are only meant to highlight the relative differences between phases.

For the purposes of reporting the structure we have used the reaction stoichiometry to define the potassium occupancy and treated the ammonia species as have the form factor of N with Hydrogen sites picked and fixed to be approximately 1Å away from N, in a similar coordination environment to that observed for the lithium analogue given in Sedlmaier et al., Journal of the American Chemical Society 2014, 136, 630–633. The occupancies of the two hydrogen sites were kept equal and fixed to give 3 H per N in the average structure. These structures are given in Tables S1, S2 and S5.

When sequentially fitting each diffraction pattern in the 0.15K + FeSe reaction we used fixed models for the initial and final products of the reaction. The models used were those taken from fitting to the diffraction patterns near the start and end of the reaction. For those models, we discounted any hydrogen and refined a mixture of N and K to occupy site *A* (without constraining the amount of K) with the form factors of O^{2-} and K^{+} chosen as a close approximation of the form factors. These structures are given in Tables S3 and S4.

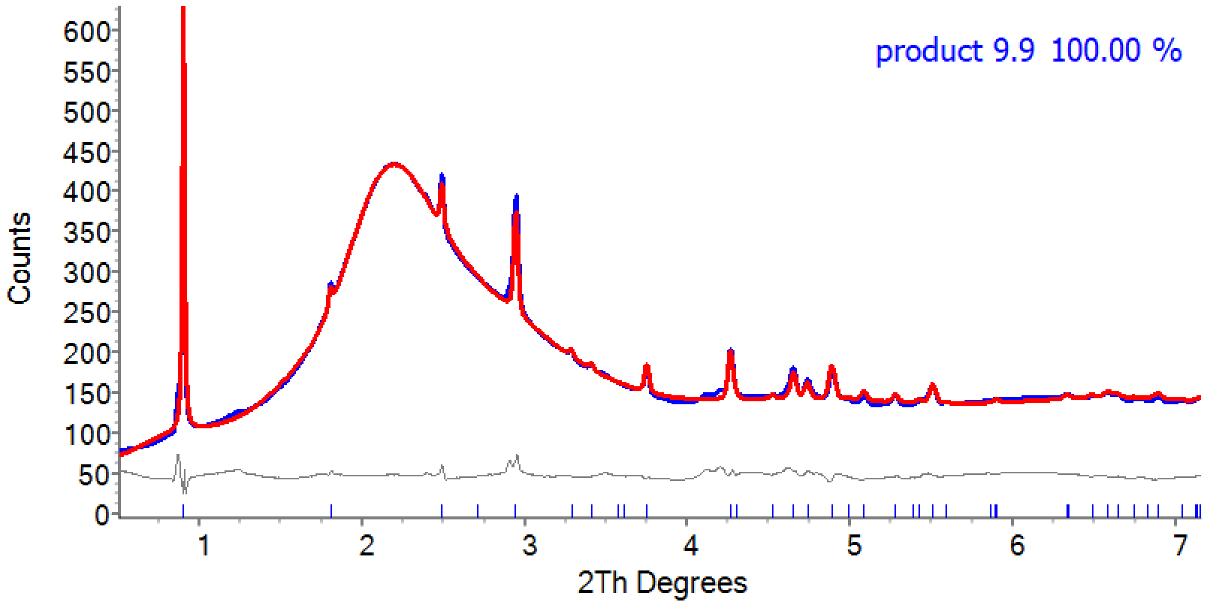

Figure S2 Rietveld refinement against the ammonia-rich final product(9.9) of the reaction of 0.3 equivalents of K with 1 equivalent of FeSe in ammonia solution. The pattern was taken at room temperature after the reaction was allowed to stir ‘offline’ for ~18 hours. λ = 0.1546 Å

Table S1 Structural parameters from the Rietveld refinement shown in Figure S2.

Space group	*P4/nmm*	origin choice	2
a / Å	3.8411(4)	*c* / Å	9.8795(8)
R_{wp} / %	2.22	V / Å^3	145.76 (3)
R_{Bragg}	1.88	Temperature / °C	22
N1-Se1 distance / Å	3.66(2)	Fe-Se distance / Å	2.426(5)
Se-Fe-Se *α* angle / °	104.7(3)	Se-Fe-Se *β* angle / °	111.9(2)

Site label	Atom type	site	x	y	z	occupancy	U_{iso} / Å^2 × 100
Fe1	Fe+2	2*a*	0.750	0.250	0	1	1.9(3)[a]
Se1	Se	2*c*	0.250	0.250	0.150(1)	1	1.9(3)[a]
NK1	N	2*c*	0.750	0.750	0.399(3)	0.70(4)[b]	1.9(3)[a]
NK1	K+1	2*c*	0.750	0.750	0.399(3)	0.3[c]	1.9(3)[a]
H1	H	8*j*	0.616[d]	0.616[d]	0.3338[d]	0.1312[d]	1.9(3)[a]
H2	H	8*j*	0.585[d]	0.585[d]	0.4456[d]	0.1312[d]	1.9(3)[a]

[a] thermal displacement parameters have all been constrained to a refine to single value
[b]occupancy of the nitrogen on the NK1 site was refined with a maximum limit of 0.7
[c]occupancy of the potassium site not refine, fixed to the value of the reaction stoichiometry.
[d]site positions and occupancies of hydrogen atoms were not refined. Occupancies were fixed to give 3 H per N and coordinates fixed to position the H atoms approximately 1Å away from N, in a similar coordination environment about N to that observed for the lithium analogue given in Sedlmaier, S. J.; Cassidy, S. J.; Morris, R. G.; Drakopoulos, M.; Reinhard, C.; Moorhouse, S. J.; O'Hare, D.; Manuel, P.; Khalyavin, D.; Clarke, S. J.. Journal of the American Chemical Society 2014, 136, 630–633.

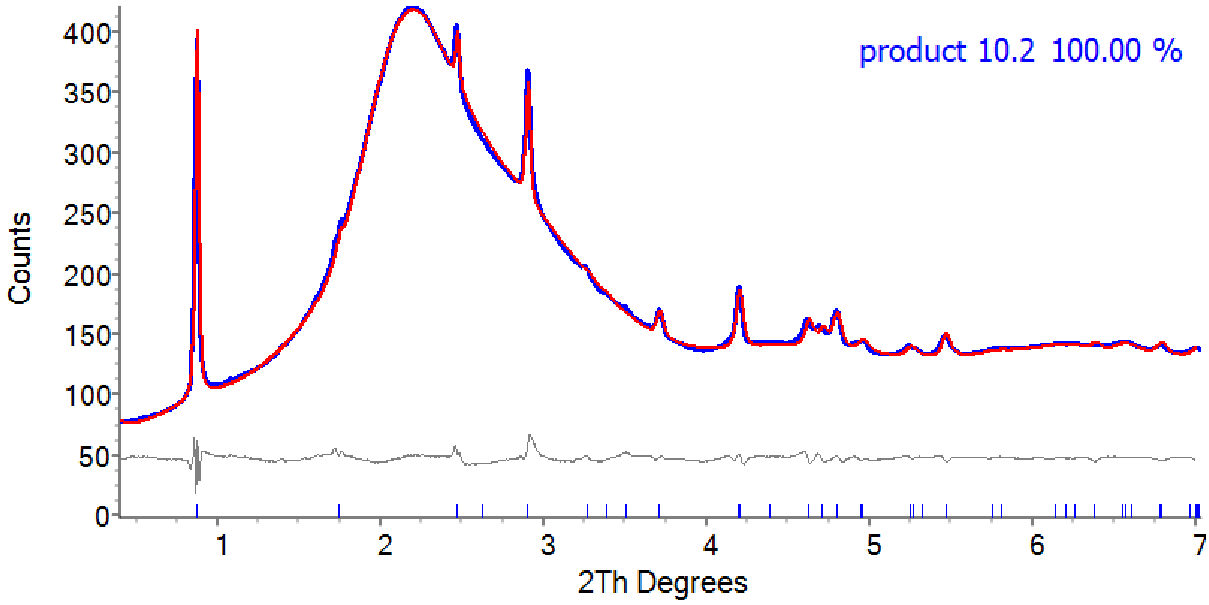

Figure S3 Rietveld refinement against the ammonia-rich final product(10.2) of the reaction of 0.15 equivalents of K with 1 equivalent of FeSe in ammonia solution. The pattern was taken at room temperature after the reaction was allowed to stir 'offline' for ~18 hours. The refined structure is given in Table S4. λ = 0.1546 Å

Table S2 Full structural parameters for the final product of the K0.15 + FeSe reaction in liquid ammonia solution.

Space group			*P*4/*nmm*	origin choice		2	
a / Å			3.8610(4)	*c* / Å		10.1890(9)	
R_{wp} / %			1.34	Volume / Å^3		151.89(4)	
R_{Bragg}			0.83	Temperature / °C		22	
N1-Se1 distance / Å			3.80(2)	Fe-Se distance / Å		2.455(6)	
Se-Fe-Se α angle / °			103.7(3)	Se-Fe-Se β angle / °		112.4(2)	
Site Label	Atom	site	x	y	z	occupancy	U_{iso} / Å^2 × 100
Fe1	Fe+2	2*a*	0.750	0.250	0	1	1.3(3)[a]
Se1	Se	2*c*	0.250	0.250	0.149(1)	1	1.3(3)[a]
NK1	N	2*c*	0.750	0.750	0.409(3)	0.85(4)[b]	1.3(3)[a]
NK1	K+1	2*c*	0.750	0.750	0.409(3)	0.15[c]	1.3(3)[a]
H1	H	8*j*	0.616[d]	0.616[d]	0.3438[d]	0.31875[d]	1.3(3)[a]
H2	H	8*j*	0.585[d]	0.585[d]	0.4556[d]	0.31875[d]	1.3(3)[a]

[a] thermal displacement parameters have all been constrained to a refine to single value
[b]occupancy of the nitrogen on the NK1 site was refined with a maximum limit of 0.85
[c]occupancy of the potassium site not refine, fixed to the value of the reaction stoichiometry.
[d]site positions and occupancies of hydrogen atoms were not refined. Occupancies were fixed to give 3 H per N and coordinates fixed to position the H atoms approximately 1Å away from N, in a similar coordination environment about N to that observed for the lithium analogue given in Sedlmaier, S. J.; Cassidy, S. J.; Morris, R. G.; Drakopoulos, M.; Reinhard, C.; Moorhouse, S. J.; O'Hare, D.; Manuel, P.; Khalyavin, D.; Clarke, S. J.. Journal of the American Chemical Society 2014, 136, 630–633.

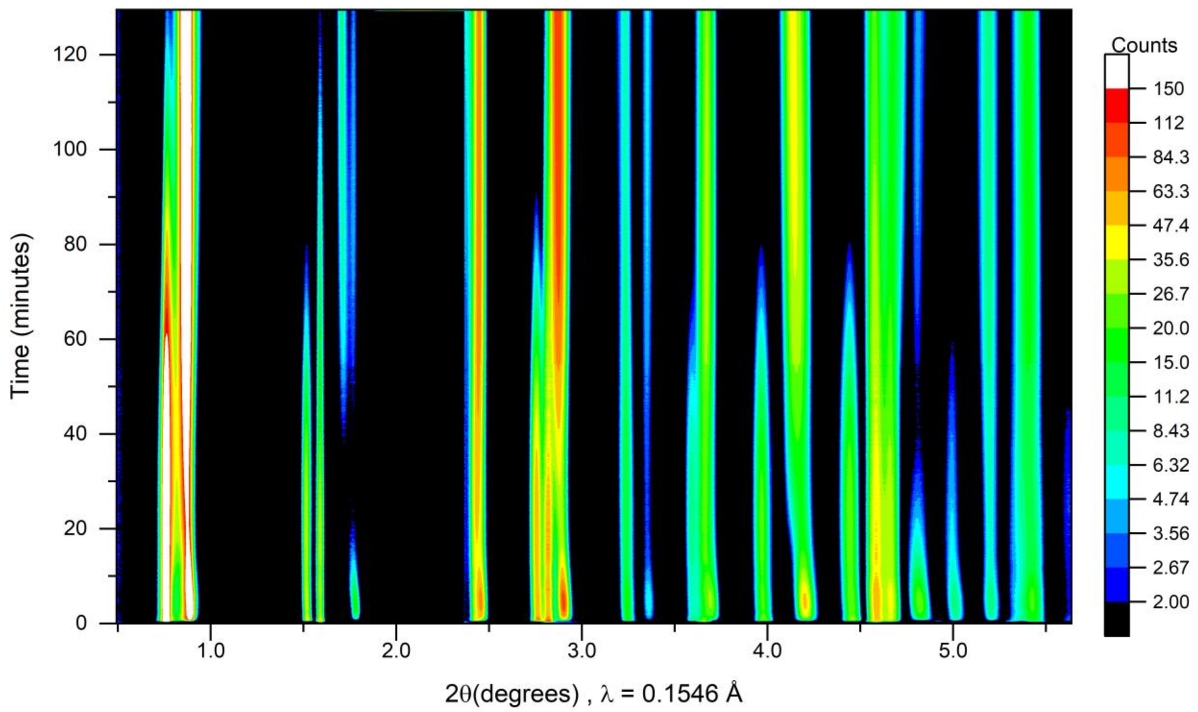

Figure S4 replot of figure 2 in two theta degrees instead of Q-value

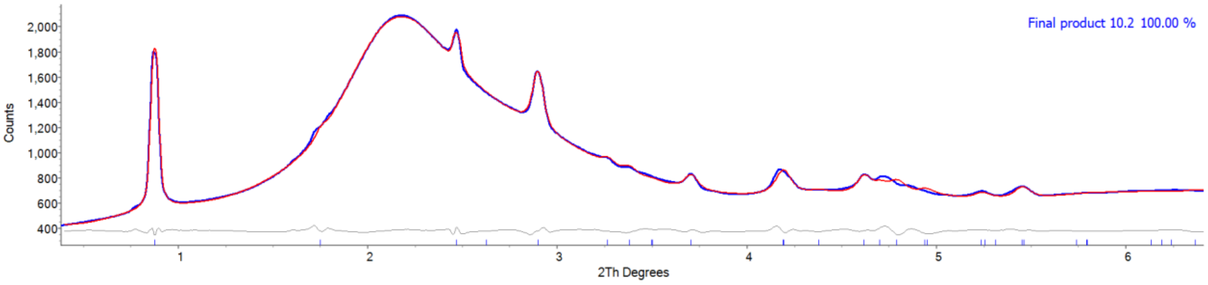

Figure S5 Rietveld refinement against the ammonia-rich final product(10.2) of the reaction of 0.15 equivalents of K with 1 equivalent of FeSe in ammonia solution. The pattern was taken at the end of the reaction time = 130 minutes. The refined structure as given in Table S2 was used as the fixed 'final product (10.2)' component in the sequential fitting of this reaction at all time points. λ = 0.1546 Å

Table S3 Full structural parameters for the refined phase of the last sequential diffraction pattern taken in the K0.15 + FeSe reaction in liquid ammonia solution shown in Figure S4. This structure was used as a fixed phase component for the sequential fitting of 'final product 10.2' across all the time points in the K0.15 + FeSe reaction as described in the main text.

**Space group**			*P4/nmm*		**origin choice**	2	
***a* / Å**			3.83764(8)		***c* / Å**	10.116(2)	
**R_{wp} / %**			1.03		**Volume / Å^3**	151.83(3)	
**R_{Bragg}**			0.29		**Temperature / °C**	~25	
**Atom Label**	**Atom type**	**site**	**x**	**y**	**Z**	**occupancy**	**U_{iso} / Å^2 × 100**
Fe1	Fe+2	2*a*	0.750	0.250	0	1	0.8(3)^a
Se1	Br-1^b	2*c*	0.250	0.250	0.150(1)	1	0.8(3)^a
NK1	O-2^b	2*c*	0.750	0.750	0.415(2)	0.68(4)^c	0.8(3)^a
NK1	K+1	2*c*	0.750	0.750	0.415(2)	0.32(4)^c	0.8(3)^a

^{a} thermal displacement parameters have all been constrained to a refine to single value
^{b}the scattering factor of Br^- and O^{2-} have been used to approximate the scattering factors of Se^{2-} and NH_3, respectively.
^{c}the occupancy of the N1 and K1 sites were constrained to be equal to a total of one.

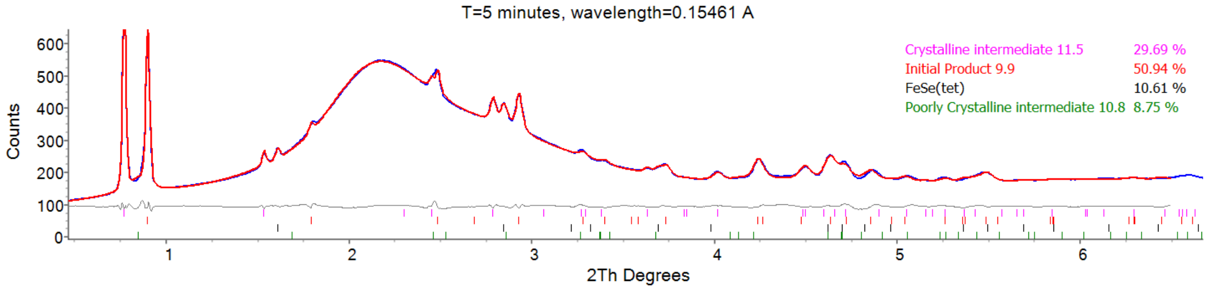

Figure S6 Rietveld refinement against the multiple phases occurring in the reaction of 0.15 equivalents of K with 1 equivalent of FeSe in ammonia solution at 5 minutes into the reaction. The temperature for the pattern was not well determined between -60 and 20 °C. Rietveld parameters for the Initial product 9.9 in this reaction were extracted from this fit, and used for subsequent fitting to the K0.15 + FeSe reaction, across all time-points as described in the main text. The refined phase is given in Table S4. λ = 0.1546 Å

Table S4 Full structural parameters for the initial product 9.9 in the K0.15 + FeSe reaction in liquid ammonia solution at 5 minutes into the reaction (shown in Figure S5). This structure was used as a fixed phase component for the sequential fitting of 'initial product 9.9' across all the time points in the K0.15 + FeSe reaction as described in the main text.

**Space group**		*P4/nmm*		**origin choice**		2	
***a* / Å**		3.8205(5)		***c* / Å**		9.8796(9)	
**R_{wp} / %**		1.16		**Volume / Å^3**		144.57(3)	
**R_{Bragg} / %**		0.21		**Temperature / °C**		~-60	
**Site Label**	**Atom type**	**site**	**x**	**y**	**z**	**occupancy**	**U_{iso} / Å^2 × 100**
Fe1	Fe+2	2*a*	0.750	0.250	0	1	1.9(3)[a]
Se1	Br-1[b]	2*c*	0.250	0.250	0.159(1)	1	1.9(3)[a]
NK1	O-2[b]	2*c*	0.750	0.750	0.414(2)	0.33(6)[c]	1.9(3)[a]
NK1	K+1	2*c*	0.750	0.750	0.414(2)	0.66(6)[c]	1.9(3)[a]

[a] thermal displacement parameters have all been constrained to a refine to single value
[b]the scattering factor of Br^- and O^{2-} have been used to approximate the scattering factors of Se^{2-} and NH_3, respectively.
[c]the occupancy of the N1 and K1 sites were constrained to be equal to a total of one.

Discussion S2: Comparison of a fitting models to the 0.15K:FeSe reaction in liquid ammonia in-situ diffraction data

As described in the main paper, it is not possible to definitively say whether the initial product(9.9) converts to the final product(10.2) via a continual change expansion in lattice parameters or whether these are two distinct phases with a phase gap between them. To model them as a single phase the lattice parameters and site occupancies are allowed to vary, while modelling them with two distinct phases can be done by fixing the lattice parameters and site occupancies of the initial product to using refined values from the 2-minute mark, and the final product using refined values at 130 minutes. Figure S6 shows that a single-phase model gives a superior Rwp in the first 60 minutes, with a particular improvement in the 5-25 minute region during which the greatest transition from one phase to another occurs. Despite this, we choose to interpret the data as there being two distinct phases because:

- The Rwp of the two-phase model is still reasonable and it contains fewer refining parameters. A better fit to the data than the single-phase model provides can easily be achieved by allowing some refinement of the two phases lattice and structural parameters to account for reasonable changes temperature and slight compositional variation in the two phases either side of the phase gap.
- That the initial product(9.9) of this reaction is indistinguishable from the product of the reaction between 0.3 K + FeSe in ammonia indicates this is a stable composition that is that this the two levels of interlayer separation,
- It is clear that the crystalline intermediate(11.5) reforms during the time the product interlayer spacing is expanding, indicating that it is an intermediary for the expansion. The behaviour of the product peak(s) is consistent with a kinetic product(9.9) being transformed into a thermodynamic product(10.2) through a reactive intermediate(11.5): growing in the initial stages, then falling as the intermediate reforms, then growing again as the thermodynamic product emerges.

-

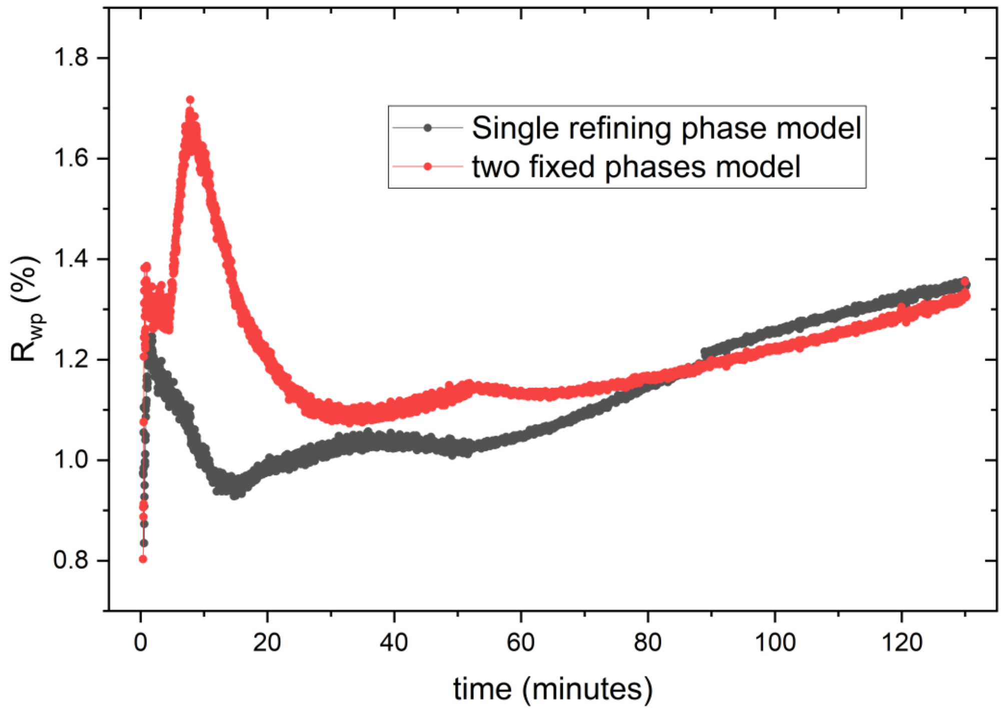

Figure S7 Rwp agreement factor versus time comparison for two alternative interpretations of the 0.15K:FeSe reaction in liquid ammonia insitu diffraction data.

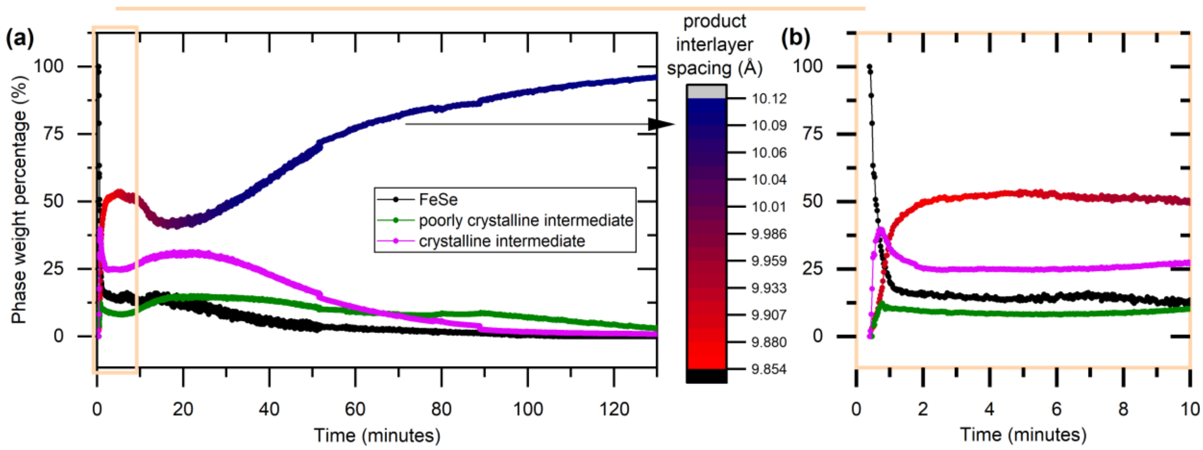

Figure S8 Weight percentages of crystalline reactants and products in the reaction as extracted from the Rietveld refinements. Analogous to Figure 4 of the main paper but using the single phase model for the product as described above.

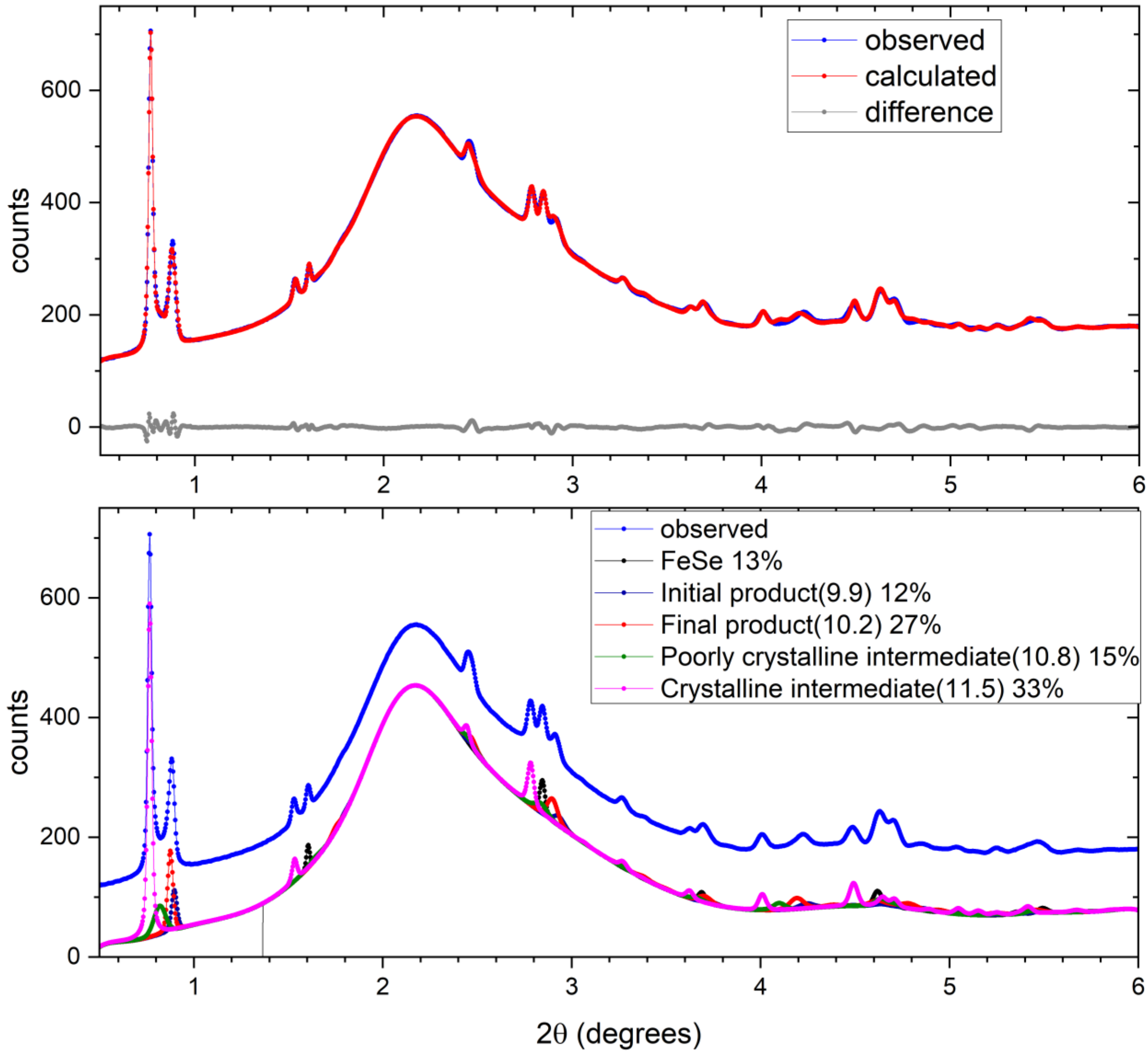

Figure S9 Rietveld refinement against the multiple phases occurring in the reaction of 0.15 equivalents of K with 1 equivalent of FeSe in ammonia solution at time point 20 minutes. The temperature for the pattern was not well determined between -60 and 20 °C. Rietveld parameters for the crystalline intermediate phase are extracted below in Table S5. The models for the initial and final products were fixed to the values in tables S3 and S2 respectively. λ = 0.1546 Å

Table S5 Full structural parameters for the crystalline intermediate 11.5 of the K0.15 + FeSe reaction in liquid ammonia solution.

**Space group**	*P*4/*nmm*	**origin choice**	2
***a* / Å**	3.8146(6)	***c* / Å**	11.5569(9)
**R_{wp} / %**	1.29	**Volume / Å^3**	168.11(6)
**R_{Bragg}**	0.31	**Temperature / °C**	-60 < T < 20
**Fe-Se distance / Å**	2.420(7)	**N1-Se1 distance / Å**	3.75(3)
**Se-Fe-Se α angle / °**	104.0(4)	**Se-Fe-Se β angle / °**	112.2(2)
**N1-K2 distance / Å**	2.54(3)		

**atom**	**site**	**x**	**y**	**z**	**occupancy**	**U_{iso} / Å^2 × 100**
Fe1	2*a*	0.750	0.250	0	1	5.4(3)[a]
Se1	2*c*	0.250	0.250	0.130(1)	1	5.4(3)[a]
N1	2*c*	0.750	0.750	0.368(5)	1[b]	5.4(3)[a]
K2	2*b*	0.750	0.250	0.5	0.22(3)	5.4(3)[a]
H1	8*j*	0.616[c]	0.616[c]	0.309[c]	0.1875[c]	5.4(3)[a]
H2	8*j*	0.575[c]	0.575[c]	0.406[c]	0.1875[c]	5.4(3)[a]

[a] thermal displacement parameters have all been constrained to a refine to single value
[b]occupancy of the nitrogen site fixed to one
[d]site positions and occupancies of hydrogen atoms were not refined. Occupancies were fixed to give 3 H per N and coordinates fixed to position the H atoms approximately 1Å away from N, in a similar coordination environment about N to that observed for the lithium analogue given in Sedlmaier, S. J.; Cassidy, S. J.; Morris, R. G.; Drakopoulos, M.; Reinhard, C.; Moorhouse, S. J.; O'Hare, D.; Manuel, P.; Khalyavin, D.; Clarke, S. J.. Journal of the American Chemical Society 2014, 136, 630–633.

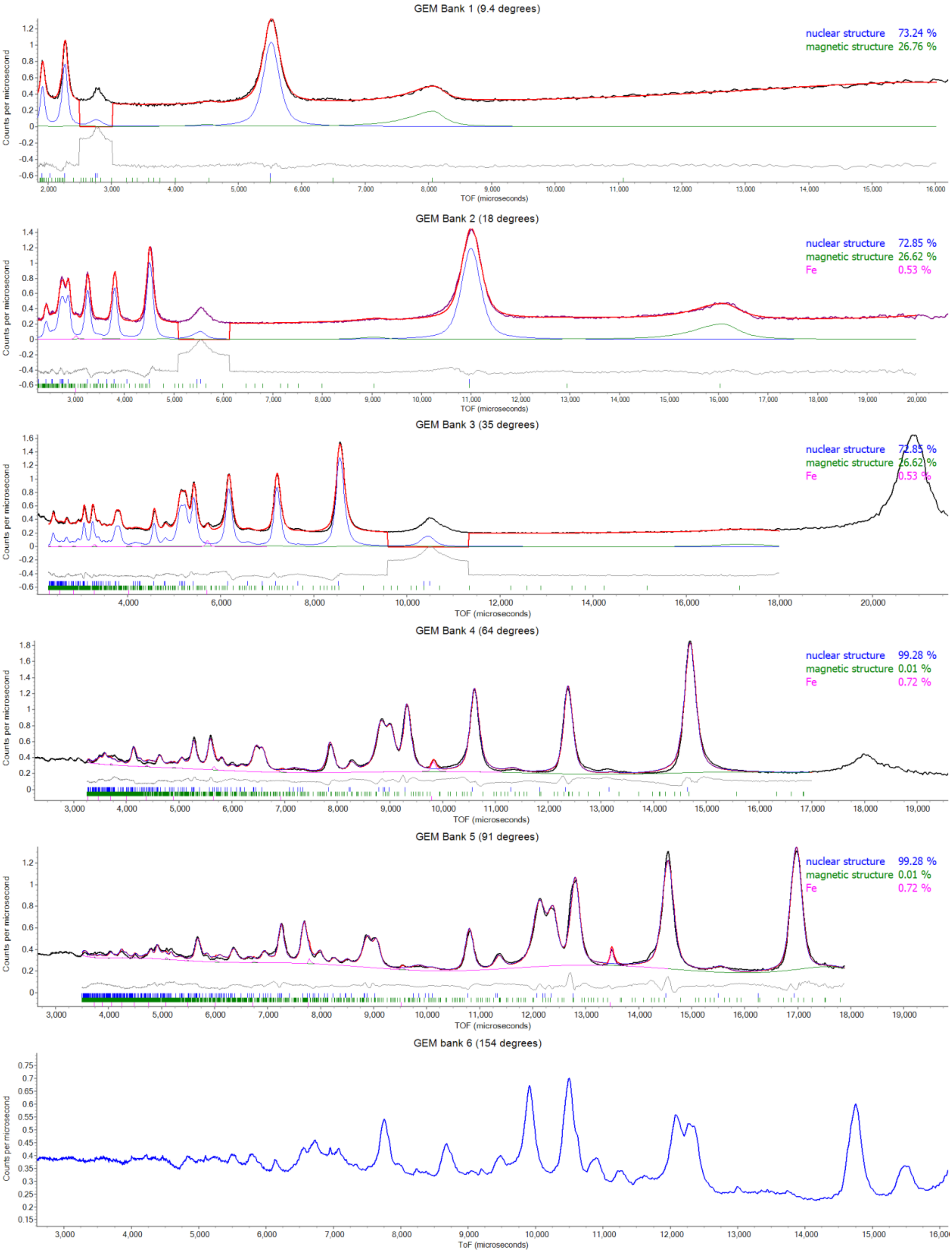

Figure S10 Rietveld against neutron powder diffraction from banks 1-5 of the GEM diffractometer for K0.3(ND2.6)0.14FeSe(7.4). It is unclear whether there is diffuse scattering underneath the peak at 10250 microseconds in bank 3 (also observed in banks 1 and 2 at different ToF), this peak required a broad raised background below it to properly fit with the nuclear model. This broad region's origin is unclear and could be magnetic, so it was decided to exclude this peak from the refinement in each bank. It was decided not to include GEM's bank 6 because it contained no additional peaks or increased resolution compared to bank 5 (resolution was sample limited). The broad peaks and a featured background meant that the

refinement of bank 6 was easily biased by background terms. Iron was not included in the refinement against bank 1 due to no peaks from those phases being present in this range.

Table S6 Parameters obtained from the Rietveld refinement of $K_{0.3}(ND_{2.6(3)})_{0.14(2)}FeSe$, corresponding to the fit shown in Figure S10

**Temperature / K**	295	***a* / Å**	3.8429(4)
**Space group**	*I*4/*mmm*	***c* / Å**	14.689(2)
**R_{wp} / %**	4.60	***V* / Å^3**	216.93(6)
**N-D distance / Å**	0.96(5)	**K-Se distance / Å**	3.454(2)
**Fe-Se distance / Å**	2.462(2)	**Se-Fe-Se (α)angle / °**	102.6(1)
**D-Se distance / Å**	2.50(5)	**Se-Fe-Se (β)angle / °**	113.02(5)

**atom**	**site**	**x**	**y**	**z**	**Occupancy**	**U_{11} / Å^2 × 100**	**U_{33} / Å^2 × 100**
Fe	4*d*	0	0.5	0.25	1.000(7)	1.09(4)	2.9(3)
Se	4*e*	0	0	0.3556(2)	1	0.10(7)	6.4(4)
N	2*a*	0	0	0	0.27(3)	6.9(8)	5.5(2)
K	2*a*	0	0	0	0.6	6.9(8)	5.5(2)
D	16*m*	0.151(9)	0.151(9)	0.034(3)	0.089(4)	6.9(8)	5.5(2)

Discussion S3: Magnetic structure of $K_{0.3}(ND_{2.6(3)})_{0.14(2)}FeSe$

A broad peak, likely to originate from magnetic ordering, is observed in bank 1 (2θ = 9.4 °) and bank 2 (2θ = 18.0 °) at a *d*-spacing of around 10.8 Å, but no further distinct peaks of magnetic origin are observed. This magnetic reflection was indexed to a 2√2 expansion of the *a* cell parameter. ISODISTORT was used to trial different all available magnetic ordering modes in a P1 symmetry supercell with a basis of {(0,0,1),(-2,2,0),(-2,-2,0)} relating it to the nuclear cell. mDT2 and mDT3 modes were found to give a model with peak intensity at 10.8 Å. Both mode types correspond to double stripe antiferromagnetic patterns shown, with mDT2 giving moments along the *c* axis and mDT3 giving moments in the *ab* plane. Of these two choices, mDT2 gave a slightly better agreement factor (4.81 % and 4.93 %, respectively) and mDT3 gives produces anomalously high intensity to a second peak at 6.05 Å. As such, a model with an mDT2 mode for the magnetic scattering has been included in banks 1 and 2. This model can be described with a 2√2 √2 1 expansion of the nuclear cell in orthorhombic space group 63.466 (BNS notation : Cmcm.1'_c[Cmmm], basis={(0,0,1),(1,-1,0),(2,2,0)}, origin=(0,0,0), s=4, i=8, k-active= (1/4,1/4,0)). In the mDT2 ordering scheme the measured ordered moment on Fe is 1.12(5) μ_B. It should be noted that with only a single clear magnetic reflection we do not have a high degree of confidence in the magnetic model, however this moment size is indicative of some degree of phase separation into antiferromagnetically ordered and superconducting phases that are not differentiable by NPD. It is observed that the shape of the magnetic peak is broadened asymmetrically towards lower d-spacing which is often a sign that the order associated with the peak is almost two dimensional with a has a very low correlation length in the third dimension. We do not attempt to analyse the magnetic peakshape in such a way here because the magnetic order observed is not a bulk property of the inhomogenous sample. It is modelled arbitrarily with a split pseudo-Voight peakshape. The ordering vector here isn't a special point in the Brillouin zone, so it is possible for there to be an incommensurability.

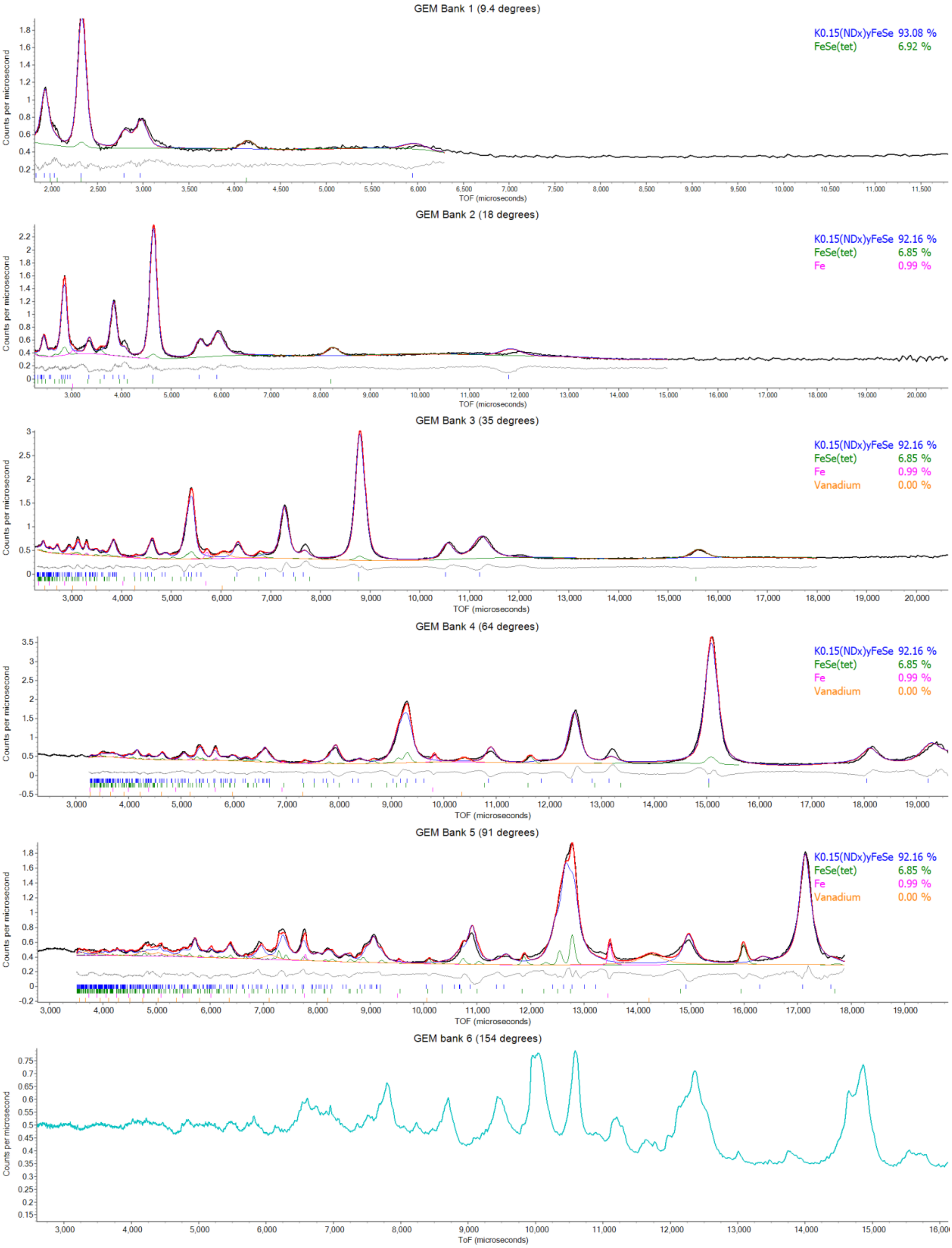

Figure S11 Rietveld against neutron powder diffraction from banks 1-5 of the GEM diffractometer for K0.15(ND2.9(3))0.35(3)FeSe(7.9). It was decided not to include GEM's bank 6 because it contained no additional peaks or increased resolution compared to bank 5 (resolution was sample limited). The broad peaks and a featured background meant that the refinement of bank 6 was easily biased by background terms. Vanadium has been included as a Pawley phase in the refinement to account for a small amount of residual scattering from the sample environment. Vanadium and iron were not included in the refinement against bank 1 due to no peaks from those phases being present in this range.

Table S7 Parameters obtained from Rietveld refinement of the $K_{0.15}(ND_{2.9(3)})_{0.35(3)}FeSe$ phase(7.9), corresponding to the fit shown in Figure S11.

**Temperature / K**	295	***a* / Å**	3.8503(3)
**Space group**	*I*4/*mmm*	***c* / Å**	15.938(3)
**R_{wp} / %**	4.76	***V* / Å^3**	236.27(6)
**N-D distance / Å**	0.96(1)	**K-Se distance / Å**	3.32(2)
**Fe-Se distance / Å**	2.407(3)	**Se-Fe-Se (*α*) angle / °**	106.2(2)
**D-Se distance / Å**	2.76(1)	**Se-Fe-Se (*β*) angle / °**	111.11(8)
**N-Se distance / Å**	3.721(3)		

**atom**	**site**	**x**	**y**	**z**	**Occupancy**	**U_{11} / Å^2 × 100**	**U_{33} / Å^2 × 100**
Fe	4*d*	0	0.5	0.25	0.98(2)	0.77(8)	5.2(5)
Se	4*e*	0	0	0.3406(3)	1	1.4(1)	4.9(5)
N	2*a*	0	0	0	0.70(5)	3.8(6)	0.0(9)
K	4*e*	0	0	0.040(6)	0.15	3.8(6)	0.0(9)
D	16*m*	0.133(2)	0.133(2)	0.0396(7)	0.254(9)	0.7(3)	0.5(1)

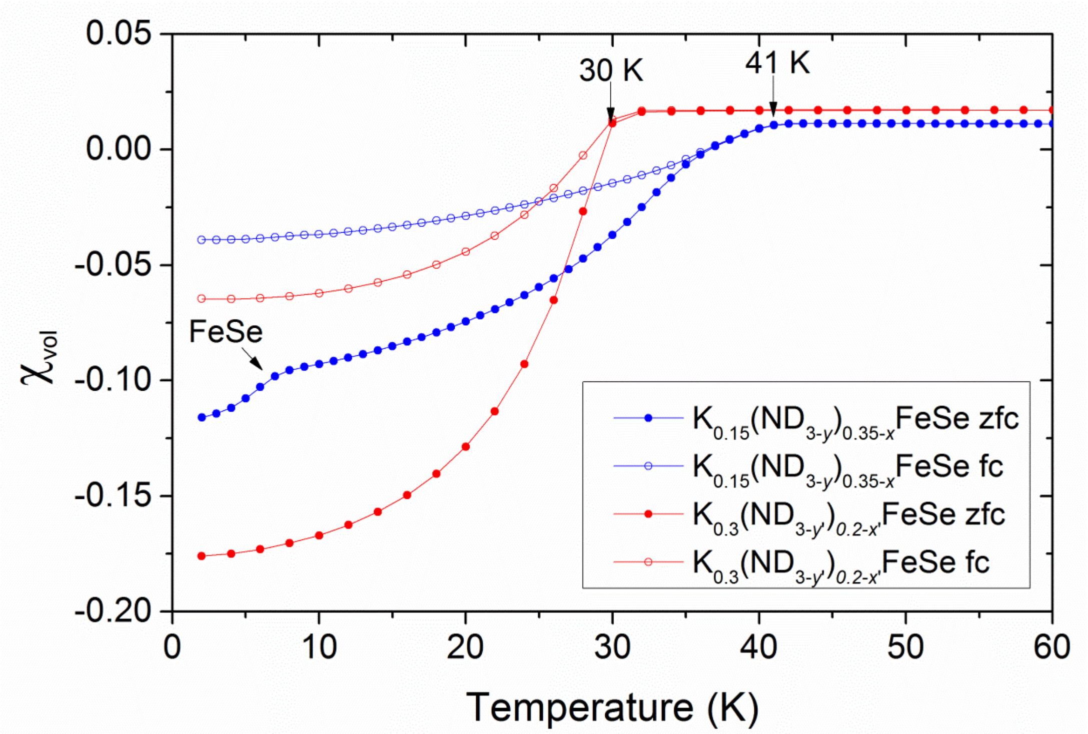

Figure S12 volume susceptibility versus tempertature plots for the deuterated ammonia-poor samples, corresponding to products 7.9 (blue) and 7.4 (red).

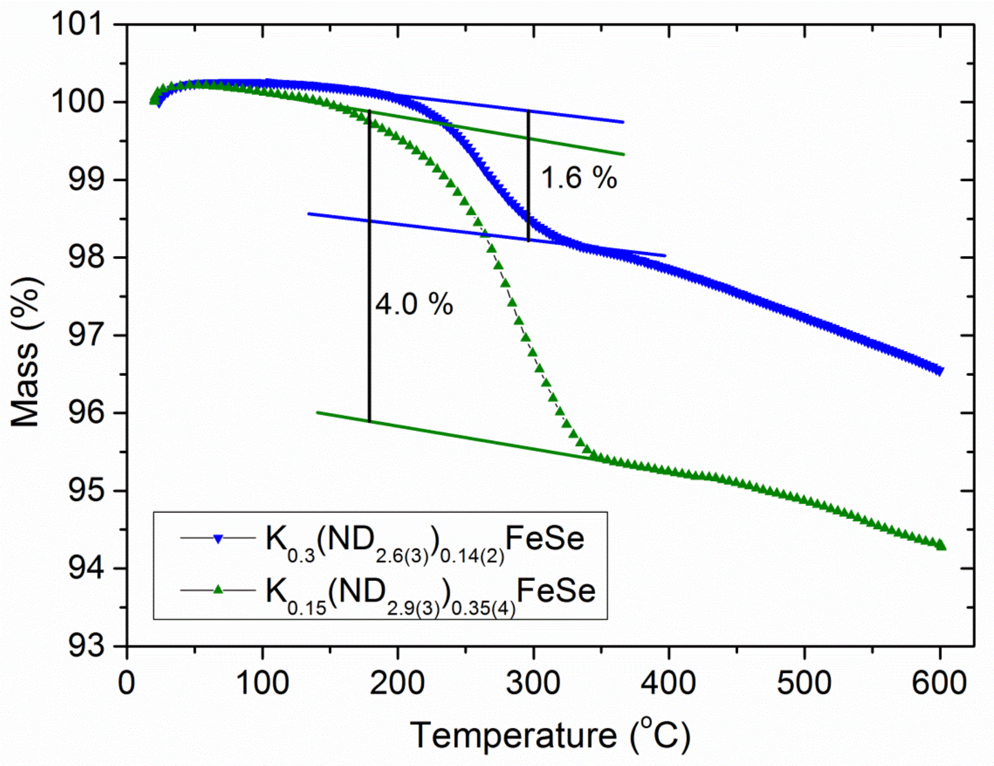

Figure S13 TGA under an argon atmosphere was carried out on the two samples used in the neutron diffraction experiment. It was necessary to expose each of the samples to air for ~ 10 seconds while transferring from an air-tight transport vessel to a pre-weighed alumina crucible on the instrument. The samples were then placed under an argon flow for the duration of the experiment. The samples were each heated from room temperature to 600 °C at a rate of 5 °C min^{-1}. A rise in the mass at the start of the measurement arises from a buoyancy effect, which remains constant after about 50 °C. A main mass loss feature occurs between 200 and 300 °C in both samples.

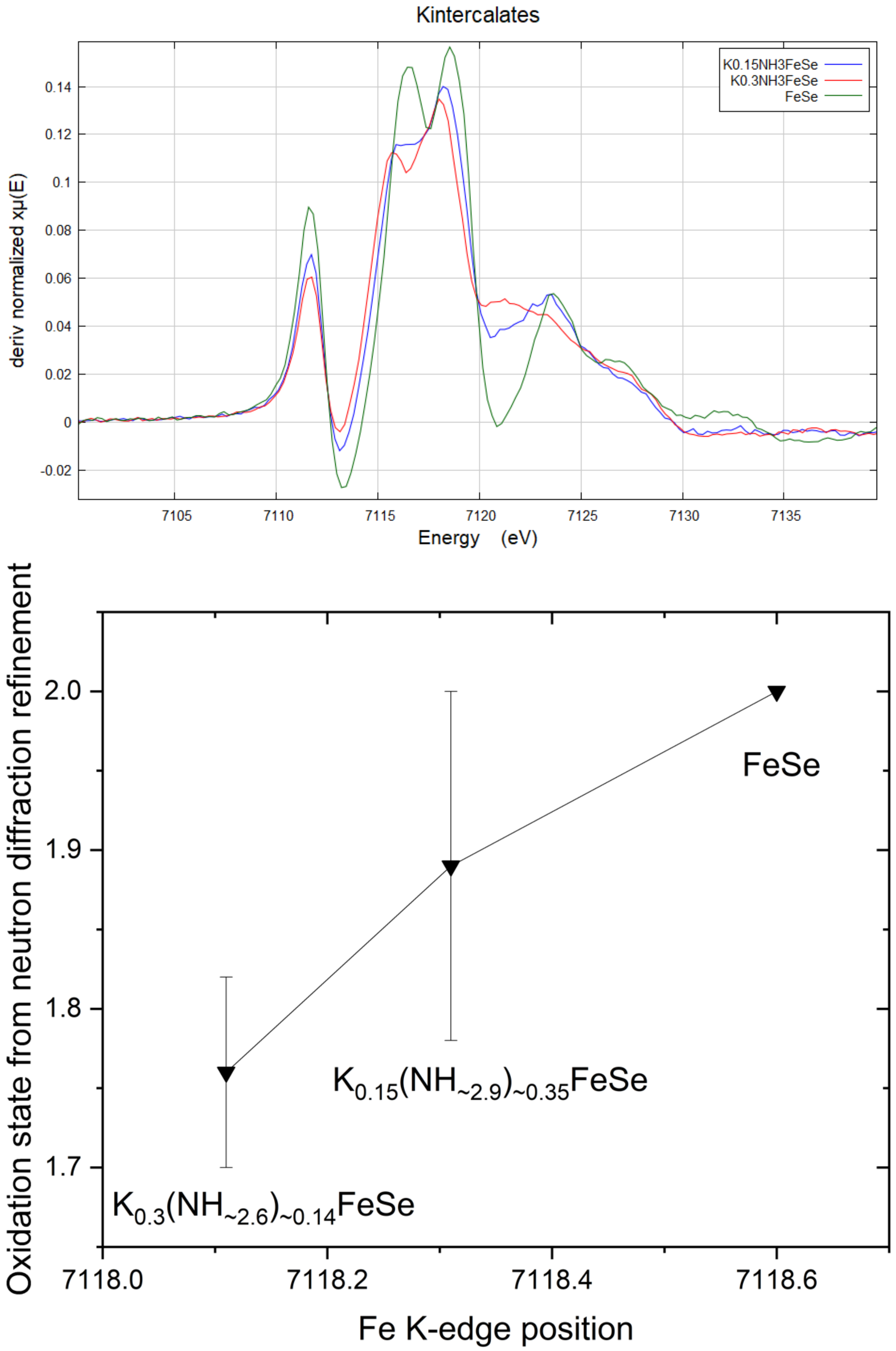

Figure S14 XANES measurements on non-deuterated samples of the (7.9) and (7.4) ammonia-poor phases confirm that there is a reductive shift in the Fe K-edge relative to the FeSe parent material for both phases, which is greater for the more potassium rich (7.4) phase. The edge positions as judged by the zero-crossing of the second derivative are 7118.60, 7118.31, and 7118.11 eV for FeSe, the $K_{0.15}NH_{\sim 2.9}FeSe$(7.9), and $K_{0.3}NH_{\sim 2.6}FeSe$(7.4), respectively. An approximately linear trend is observed between the oxidation state refined by neutron diffraction and the edge position, which adds confidence to the high uncertainties of the oxidation states assigned by neutron diffraction.

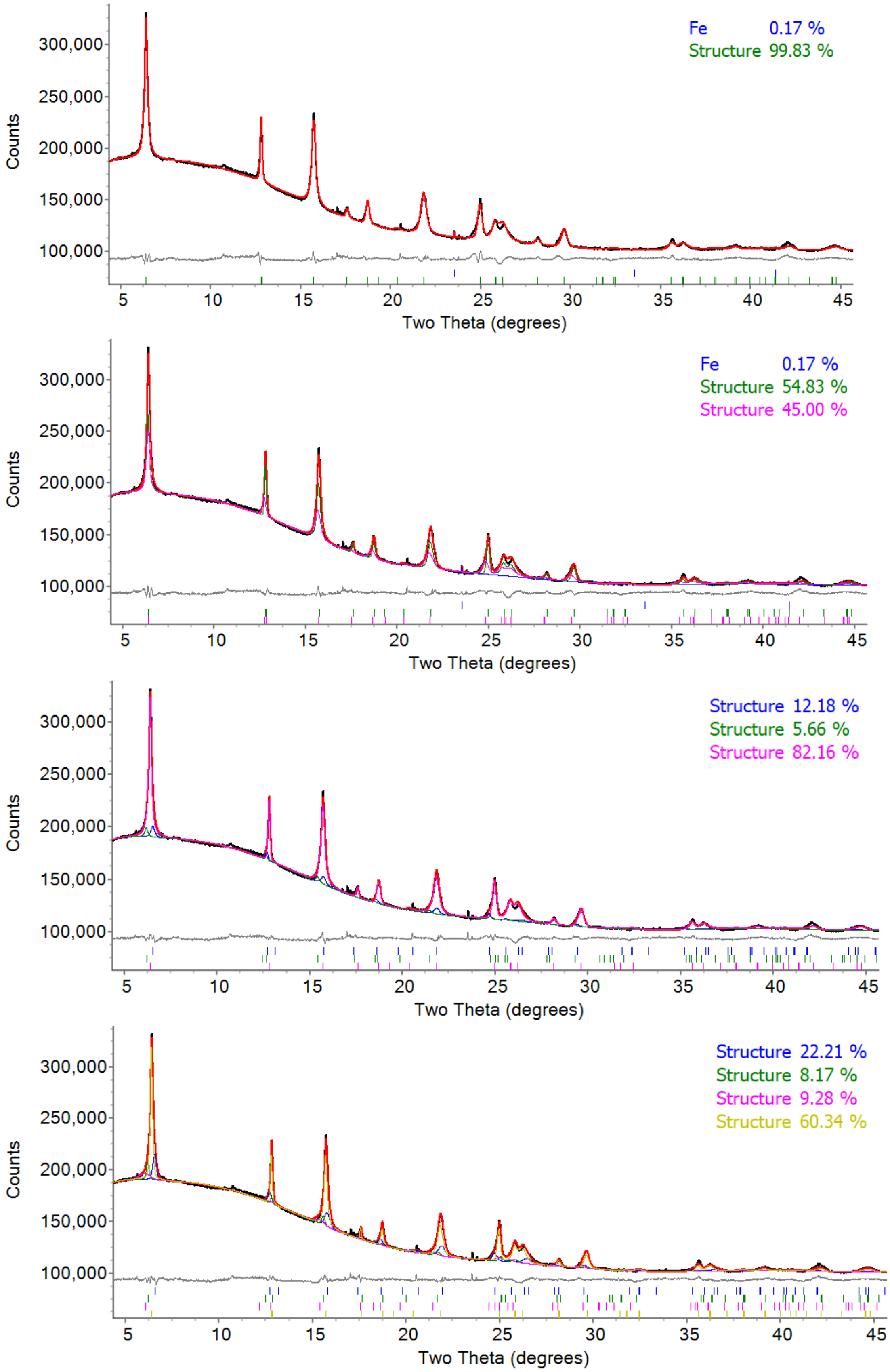

Figure S15 Rietveld refinement against SXRPD for $K_{0.3}(NH_{2.6})_{0.14}FeSe$(7.4). From top to bottom these use 1, 2,3, and 4 phase models with Rwp of 1.00, 0.91, 0.87 and 0.84 respectively. In each case the phases were constrained to have the same occupancies, fractional coordinates and thermal displacement parameters but allowed to have different peakshapes and different lattice parameters. λ=0.826989 Å

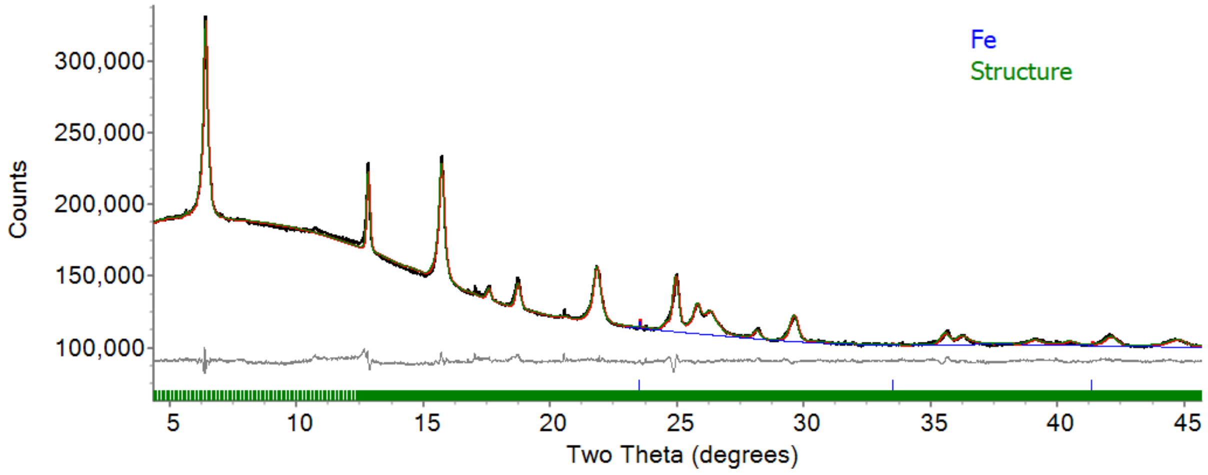

Figure S16 replot of Figure 5a in two theta rather than Q-space. Rietveld refinement against SXRPD for K0.3(NH2.6)0.14FeSe(7.4) using a 200 layer supercell of FeSe and K/N layers (R_{wp} = 0.87). λ=0.826989 Å

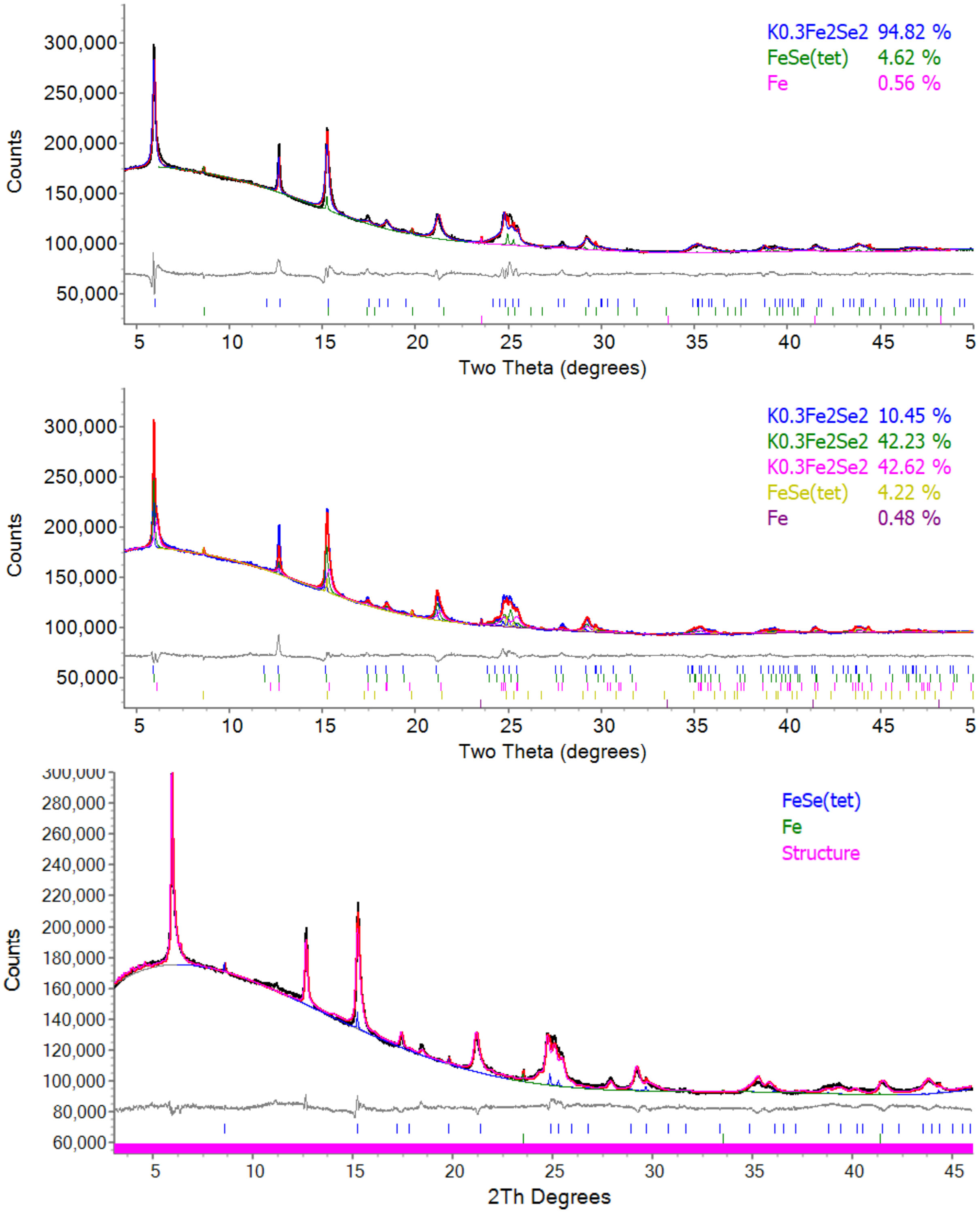

Figure S17 Rietveld refinement against SXRPD for $K_{0.15}(NH_{2.9})_{0.35}FeSe(7.9)$. From top to bottom these use 1, 3, and a 220 layer supercell model (replot of Figure 6 in two theta units) Rwp 1.51, 1.14, and 1.17. λ=0.826989 Å

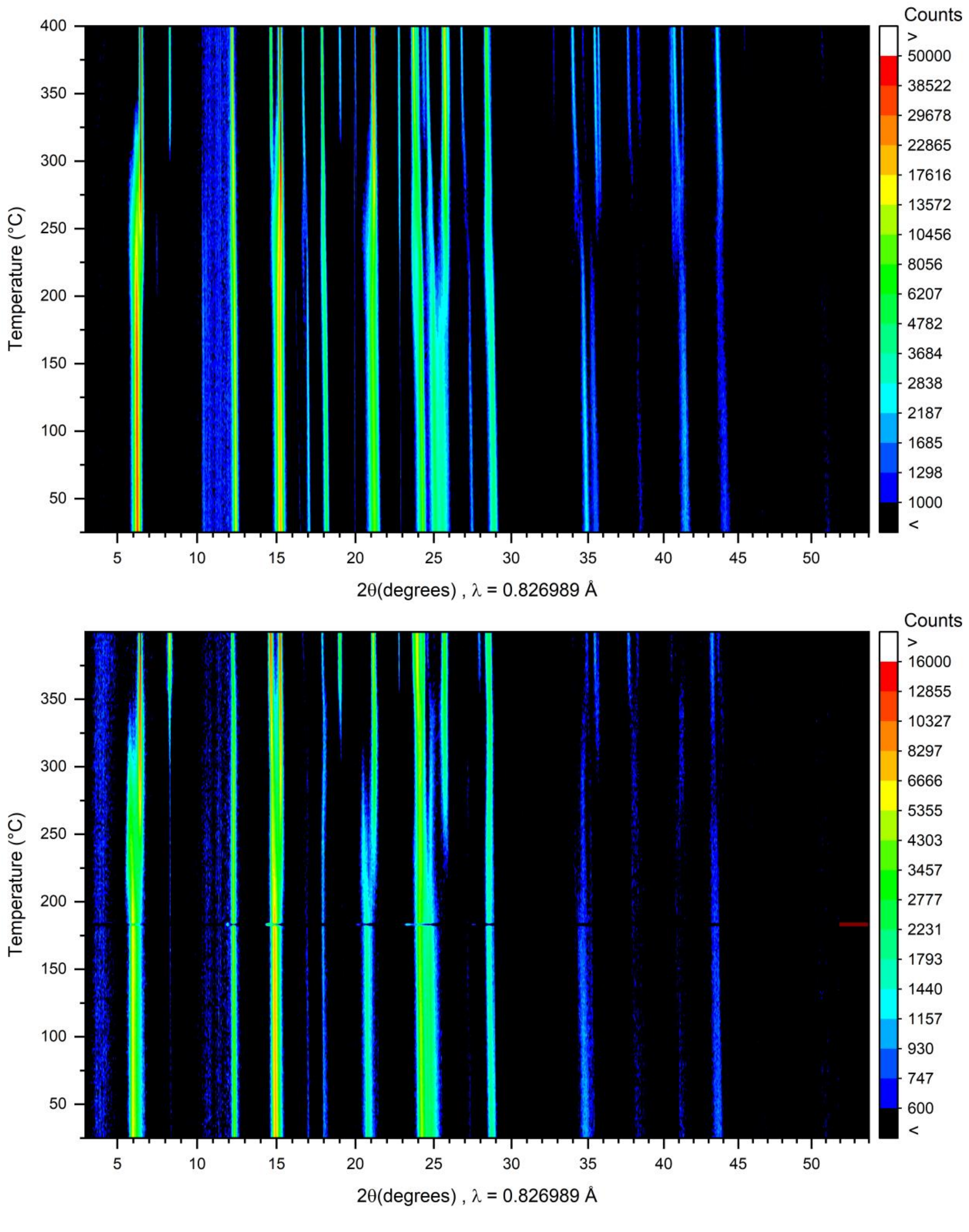

Figure S18 **Background subtracted variable temperature powder X-ray diffraction for the thermal decomposition of $K_{0.3}(NH_{\sim 2.6})_{\sim 0.14}FeSe$(7.4) (Top) $K_{0.15}(NH_{\sim 2.9})_{\sim 0.35}FeSe$(7.9) (bottom).**

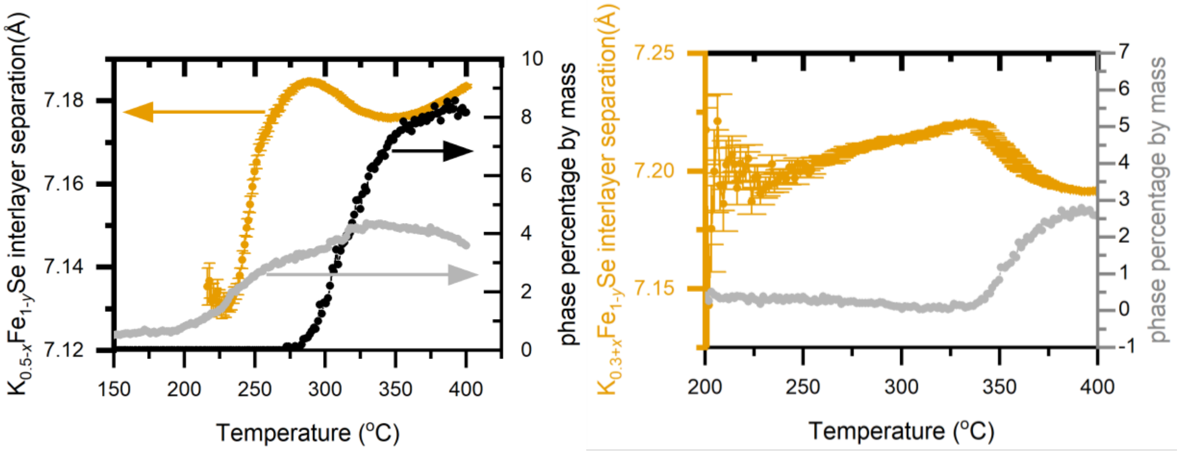

Figure S19 (left) interlayer separation (= c lattice parameter / 2) of the $K_{0.5-x}Fe_{1-y}Se$ phase that forms as a product from thermal decomposition of K0.3(NH~2.6)~0.14FeSe(7.4) during annealing plotted alongside the phase percentage of iron and iron selenide side products. (right) interlayer separation (= c lattice parameter / 2) of the $K_{0.5-x}Fe_{1-y}Se$ phase that forms as a product from thermal decomposition of K0.15(NH~2.9)~0.35FeSe(7.9) during annealing plotted alongside the phase percentage of iron side products. This is further evidence of product 7.2 forming as an iron vacancy free phase from the decomposition of product 7.9 but not from 7.4.

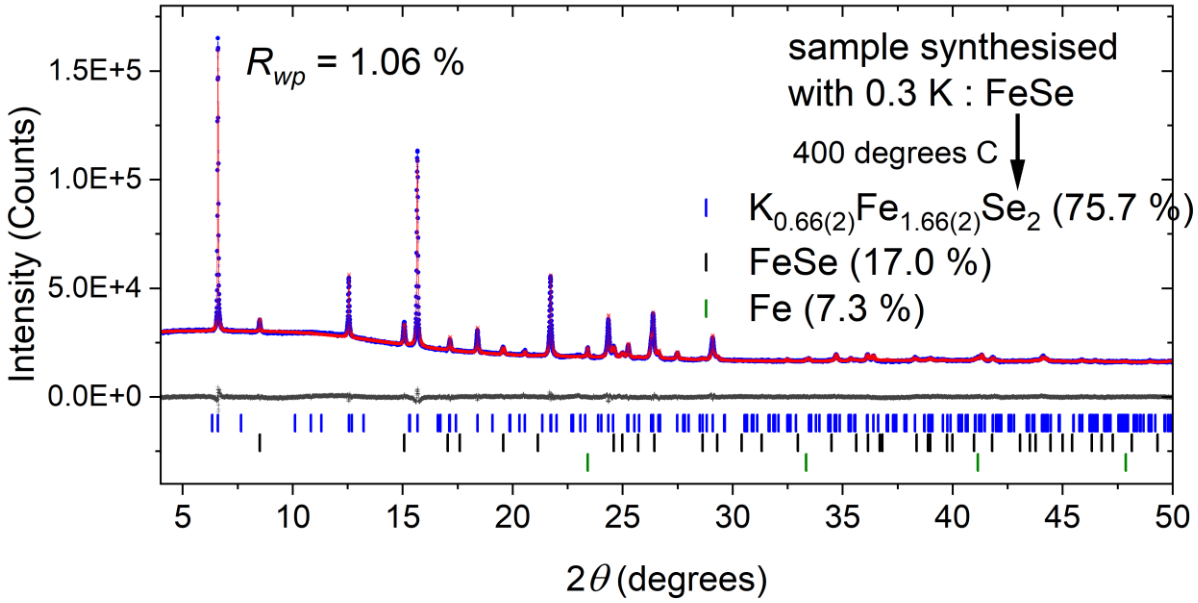

Figure S20 Rietveld refinement against the diffraction patterns of the final products of the annealing of $K_{0.3}(NH_{2.6})_{0.14}FeSe(7.4)$. Pattern taken at 400 °C, the sample capillary broke before a room temperature pattern could be taken. λ=0.826989 Å

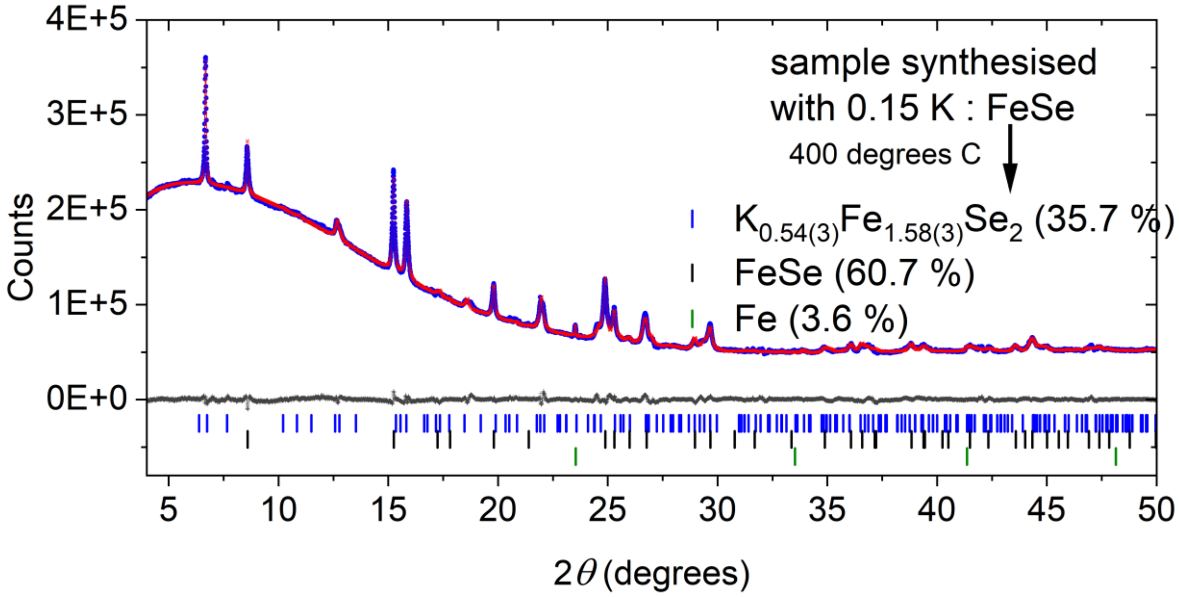

Figure S21 Rietveld refinement against the diffraction patterns of the final products of the annealing of $K_{0.15}(NH_{2.9})_{0.35}FeSe(7.9)$. Pattern taken at room temperature after heating to 400 °C. λ=0.826989 Å

Table S8 General structure of the vacancy ordered $K_xFe_{2-y}Se_2$ obtained in the high temperature synthesis from the elements,[79] and from annealing the potassium and ammonia intercalated iron selenide phases.

General structure in space group *I*4/*m*					
A	= $\sqrt{5}$ × parent *a* cell parameter		***c***	= parent *c* cell parameter	
**atom**	**site**	**x**	**y**	**z**	**Occupancy**
K1	8*h*	xK1	yK1	0.5	occK1
K2	2*b*	0	0	0.25	occK2
Fe1	16*i*	xFe1	yFe1	zFe1	occFe1
Fe2	4*d*	0	0	0.25	occFe2
Se2	16*i*	xSe1	ySe1	ZSe1	1
Se1	4*e*	0	0	zSe2	1

Table S9 Refined structural parameters from the fits in Figures S20 and S21. Fe oxidation states assume that Se is a divalent anion.

**K: FeSe ratio**	***a* / Å**	***c* / Å**	**occK1**	**occK2**	***x***	**occFe1**	**occFe2**	***2-y***	***Fe ox. state***
**0.3:1**	*	*	0.65(1)	0.71(5)	0.66(2)	0.838(8)	0.79(3)	1.66(2)	2.01(3)
**0.15:1**	8.6712(3)	14.173(1)	0.61(4)	0.23(8)	0.54(3)	0.81(2)	0.72(4)	1.58(3)	2.19(5)

*** The capillary of the annealed sample with 0.3 equivalents of potassium broke before a final collection at room temperature could be taken. Reported occupancy values are from a refinement at 400 °C, which is the refinement shown in Figure S20 The unit cell parameters of the 0.3 potassium equivalent material at 400 °C, are *a* = 8.7686(1) and *c = 14.3633(3) Å, cf.* the 0.15 potassium equivalent material at 400 °C, which has *a* =8.7530(3), *c* = 14.389(1) Å.**

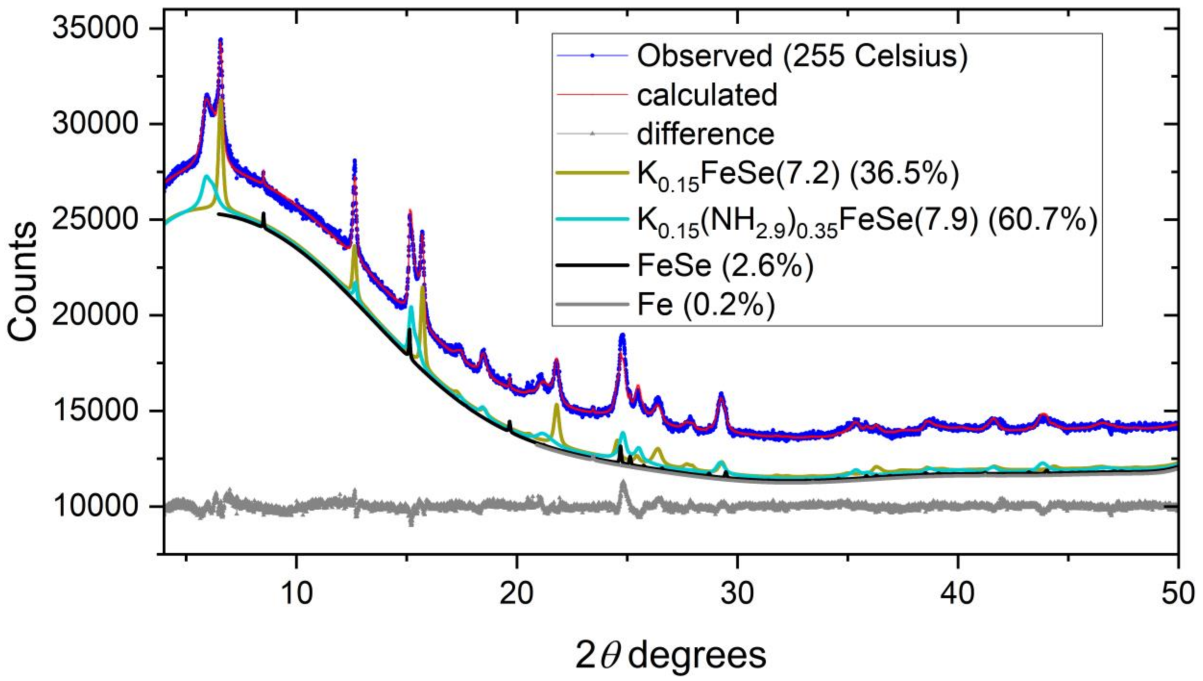

Figure S22 Rietveld refinement against the diffraction pattern of $K_{0.15}(NH_{2.9})_{0.35}FeSe$(7.9) at 255 °C taken during a continuous variable temperature sweep, corresponding to a single data point in the Figure 9(c). The structural parameters for product 7.2 in table S10 are taken from this fit. λ=0.826989 Å

Table S10 Parameters obtained from Rietveld refinement of the $K_{0.15}FeSe$ phase(7.2), corresponding to the fit shown in Figure S22.

**Temperature / K**	528			**a / Å**		3.8867(4)	
**Space group**	*I*4/*mmm*			**c / Å**		14.408(3)	
**R_{wp} / %**	1.06			**V / Å³**		217.65(6)	
**R_{Bragg} / %**	0.71						
**K-Se distance / Å**	3.420(4)			**Fe-Se distance / Å**		2.496(5)	
**Se-Fe-Se (α) angle / °**	102.2(3)			**Se-Fe-Se (β) angle / °**		113.2(2)	
**Site label**	**Atom type**	**site**	**x**	**y**	**z**	**Occupancy**	**U_{iso} / Å² × 100**
Fe	Fe+2	4*d*	0	0.5	0.25	1	9.7(6)[a]
Se	Se	4*e*	0	0	0.3587(6)	1	9.7(6)[a]
K	K+1	2*a*	0	0	0	0.47(2)	9.7(6)[a]

[a] thermal displacement parameters have all been constrained to a refine to single value

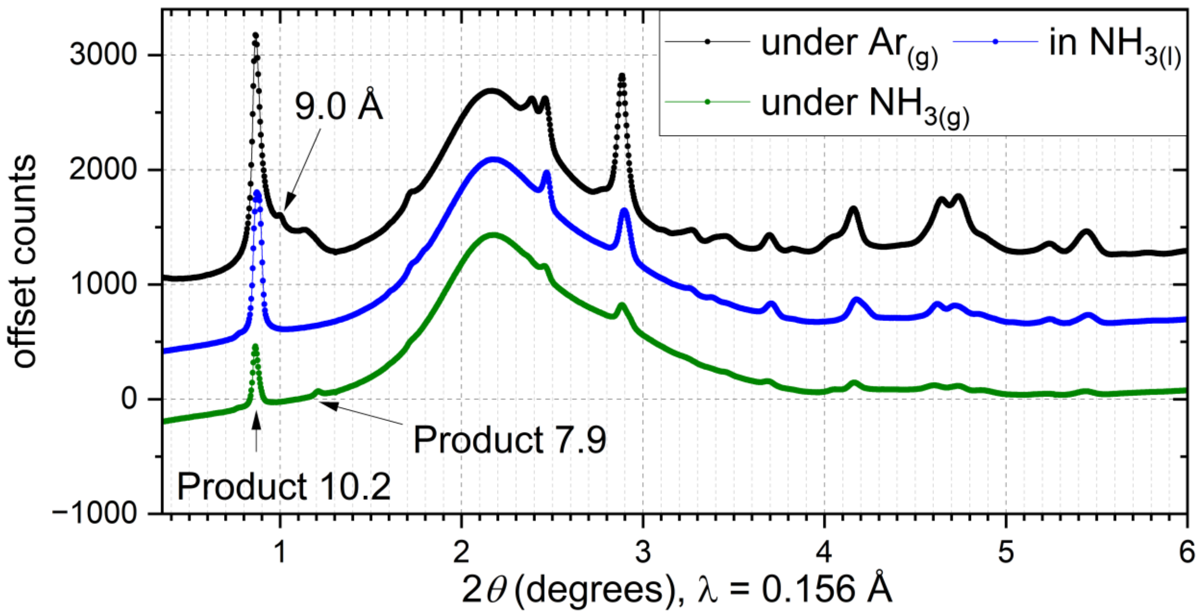

Figure S23 removal of ammonia gas from product 10.2 after the *in situ* experiment on beamline I12 at Diamond light source. The blue line shows the product phase at the end of the reaction. Measuring the sample as a function of ammonia vapour pressure was not possible with the experimental setup used but ex situ measurements were performed. The ammonia liquid was allowed to boil off at room temperature through a mercury manometer to leave the sample under approximately 1 atm of ammonia gas. We collected a snapshot of the powder in the reaction vessel at room temperature (green line) and observed that product 10.2 was mostly preserved, but a small amount of the ammonia poor product 7.9 had already formed. The liquid ammonia was allowed to boil off a second sample, but this time the sample was then exposed to a flow of dry argon gas to remove the ammonia rather than a vacuum (which causes formation of product 7.9. The snapshot of the powder in the reaction vessel after this treatment (black line) has a complex diffraction pattern that appears to show a broad transition from the ammonia rich phase to the ammonia poor, with what might be an additional intermediate phase with a peak at 9 Å. Note that differences in intensity relative to background are the result of different amounts of powder being exposed.
